# Bifacially Engineered Perovskite‐Based Synaptic Memristors Achieve High Linearity and Symmetricity for Accurate and Robust Neuromorphic Computing

**DOI:** 10.1002/advs.202511489

**Published:** 2025-08-20

**Authors:** Jang Woo Lee, Liang Cai, Jeong‐Seok Nam, Dawoon Kim, Taehoon Kim, Sihyeok Kim, Jae Ho Lee, Cheolhwa Jang, Sungpyo Baek, Jiye Han, Kiyong Kim, Seongpil An, In Chung, Eunsang Kwon, Sungjoo Lee, Il Jeon

**Affiliations:** ^1^ Department of Nano Engineering Department of Nano Science and Technology SKKU Advanced Institute of Nanotechnology (SAINT) Sungkyunkwan University (SKKU) Suwon 16419 Republic of Korea; ^2^ Research and Analytical Center for Giant Molecules Graduate School of Science Tohoku University Sendai 980‐8578 Japan; ^3^ School of Chemical and Biological Engineering and Institute of Chemical Processes Seoul National University Seoul 08826 Republic of Korea; ^4^ Department of Future Energy Engineering (DFEE) Sungkyunkwan University (SKKU) Suwon 16419 Republic of Korea; ^5^ SKKU Global Research Center (SGRC) Sungkyunkwan University (SKKU) Suwon 16419 Republic of Korea

**Keywords:** additives, linearity, memristors, metal halide perovskites, neuromorphic computing, symmetricity, synapses

## Abstract

Achieving both high linearity and symmetricity in metal halide perovskite (MHP)‐based memristors remains challenging, primarily due to their abrupt switching behaviors and irregular conductive filament (CF) pathways. Here, bifacially engineered MHP memristors exhibiting simultaneous high linearity, symmetricity, and reliability are reported. Top‐surface passivation using phenylethylammonium iodide (PEAI) facilitates the formation of an ultrathin 2D perovskite layer (PEA_2_PbI_4_), promoting gradual switching and effectively suppressing ion migration during CF formation, thereby significantly enhancing the linearity of long‐term potentiation. Meanwhile, bottom‐side PEAI treatment alleviates tensile strain and enhances perovskite grain uniformity, leading to stable CF rupture and improved linearity in long‐term depression as well as symmetricity. The resulting bifacially engineered memristor device achieves an exceptionally high *I*_on_/*I*_off_ ratio of 3.67 × 10^5^, remarkable endurance exceeding 11 000 cycles, and robust data retention time over 10^5^ s. Moreover, these bifacially engineered synaptic memristors demonstrate superior classification accuracies of 92.60% and 94.53% in Canadian Institute for Advanced Research 10 (CIFAR‐10) and Modified National Institute of Standards and Technology (MNIST) simulations, respectively. This study provides an effective engineering strategy for overcoming persistent challenges in MHP‐based memristors, thus advancing their potential for next‐generation hardware‐based neuromorphic computing applications.

## Graphical abstract

Bifacially engineering metal halide perovskite‐based memristors using phenylethylammonium iodide without oxide layers concurrently achieves high linearity, symmetricity, and reliability—essential for analog neuromorphic computing. The devices exhibit enhanced synaptic behavior, a high on/off ratio of 3.67 × 10^5^, and exceptional endurance exceeding 11 000 cycles and long retention characteristics of over 10^5^ s. CIFAR‐10 and MNIST simulations demonstrate high accuracies of 92.60% and 94.53%, respectively.

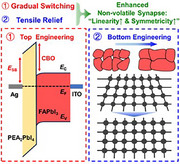

## 1 Introduction

Neuromorphic computing, inspired by the human brain, has gained significant attention due to its capability for massive parallel computation and low‐power consumption.^[^ ^1^, ^2^, ^3^ ^]^ The development of hardware‐based neuromorphic computing systems requires the implementation of analog synaptic devices with high linearity and symmetricity.^[^ ^4^, ^5^, ^6^ ^]^ Linearity is critical for precise synaptic weight (*W*) modulation, ensuring accurate learning, while symmetricity is essential for preventing *W* degradation during updates in neuromorphic computing.^[^ ^7^ ^]^ Additionally, although conceptually distinct from linearity and symmetricity, reliability—quantified through endurance and retention time—is equally crucial, as it underpins the robustness of synaptic devices.^[^ ^8^, ^9^ ^]^ Among various candidates, memristors have emerged as a promising technology for synaptic applications due to their high integration density (4F^2^), low‐power consumption, and ease of fabrication.^[^ ^10^, ^11^, ^12^ ^]^ However, conventional memristors face critical challenges, including nonlinearity, asymmetric weight updates, and suboptimal reliability, which hinder their practical implementation in neuromorphic computing systems.^[^ ^7^, ^9^, ^13^, ^14^ ^]^ Addressing these limitations is essential for the advancement of robust and efficient memristor‐based neuromorphic architectures.

Over the past decade, metal halide perovskites (MHPs) have garnered considerable attention in optoelectronics due to their exceptional properties, including high carrier mobility and solution processability at room temperature.^[^ ^15^, ^16^, ^17^ ^]^ However, their large sweep‐dependent current–voltage (*I–V*) hysteresis and ion migration‐driven degradation have posed significant challenges for optoelectronic applications. Interestingly, these same limitations present advantages for memristor applications, positioning MHP‐based memristors as a potential breakthrough for advancing the field of MHPs.^[^ ^18^, ^19^, ^20^, ^21^, ^22^, ^23^ ^]^ The *I–V* hysteresis behavior in MHP memristors translates to a desirably high on/off‐state current (*I*_on_/*I*_off_) ratio, while the low energy barrier for ion migration hints at fast switching and sub‐1‐V operation.^[^ ^18^, ^24^, ^25^, ^26^, ^27^, ^28^, ^29^, ^30^, ^31^ ^]^ MHP‐based memristors offer a potential solution to the intrinsic nonlinearity and asymmetricity challenges in conventional memristors, as the energy barrier heights and grain morphology of MHPs can be tailored via structural and chemical modifications during fabrication.^[^ ^6^, ^26^, ^31^ ^]^ While MHP memristors still suffer from nonlinearity arising from the abrupt switching in *I–V* characteristics primarily caused by the low ion migration energy between the MHP layer and the electrode,^[^ ^23^, ^32^ ^]^ introducing a high bandgap or a conduction band offset (CBO) can suppress the abrupt switching behavior and thereby enhance linearity.^[^ ^32^ ^]^ However, increasing the energy barrier height to improve linearity often compromises reliability.^[^ ^21^ ^]^ Additionally, the presence of irregular filamentary conduction pathways, stemming from small and nonuniform MHP grains, contributes to poor symmetricity, reduced reliability, and a lower *I*_on_/*I*_off_ ratio.^[^ ^6^, ^18^, ^20^, ^22^ ^]^ Specifically, poor symmetricity—mainly stemming from the pronounced nonlinearity during long‐term depression (LTD)—makes it particularly difficult to achieve linear and balanced updates between long‐term potentiation (LTP) and LTD, posing a key challenge in the development of MHP‐based memristors.^[^ ^6^, ^7^, ^32^, ^33^, ^34^, ^35^ ^]^ Despite extensive research efforts by many research groups, only a few reported cases have successfully demonstrated high linearity and symmetricity in MHP memristors, but still with critical limitations.^[^ ^6^, ^32^ ^]^ For example, Kim et al. demonstrated a pure 2D MHP‐based memristor for a synapse showing both high linearity and symmetricity.^[^ ^6^ ^]^ However, the device still presents limitations in terms of *I*_on_/*I*_off_ ratio and long‐term reliability. To address the issue of nonlinearity, Lee et al. proposed a bilayer structure incorporating an atomic‐layer‐deposited SnO_2_ with an MHP‐based memristor.^[^ ^32^ ^]^ Nonetheless, severe asymmetricity is observed in their structure, and the benefit from the CBO is limited because the adapted SnO_2_ layer does not possess an ideal energy level.^[^ ^36^, ^37^, ^38^ ^]^ Furthermore, introducing oxide layers involves a cumbersome fabrication step and is applicable only as the bottom layer. In both cases, due to the direct metal–perovskite contact, the proposed structures remain limited by poor reliability.^[^ ^22^, ^39^, ^40^ ^]^ Thus, an ideal MHP memristor strategy should provide high linearity, symmetricity, reliability, and a high *I*_on_/*I*_off_ ratio using a facile, low‐cost fabrication process without additional oxide layers.

In this work, we demonstrate MHP memristors that simultaneously achieve high linearity, symmetricity, and reliability with a high *I*_on_/*I*_off_ ratio by employing a bifacial engineering strategy. Introducing phenylethylammonium iodide (PEAI) above and below the MHP active layer formed a protective energy barrier and tensile strain reliever, respectively. Forming an ultrathin hydrophobic 2D perovskite layer on top of a 3D MHP layer introduced an energy barrier, which includes a Schottky barrier (SB) and CBO; the former contributes to hindering ion migration from the metal, while the latter contributes more greatly to gradual switching behavior. In other words, linearity is enhanced without the reliability compromise. This top‐side treatment predominantly improves the linearity of LTP, with limited impact on the linearity of LTD. In contrast, PEAI treatment on the bottom side increased the size and quality of the perovskite grain, which regulated the conductive filaments (CFs). This leads to significant improvements in the linearity of LTD rather than that of LTP, as well as symmetricity and reliability while maintaining a high *I*_on_/*I*_off_ ratio. Therefore, bifacial engineering, which shows a synergistic effect, is essential to concurrently achieve both high linearity and high symmetricity. The effects of the bifacial engineering of PEAI are systematically investigated by conducting various material and device characterizations on four distinct configurations: no PEAI treatment, top‐only PEAI treatment, bottom‐only PEAI treatment, and bifacial PEAI treatments. Ultimately, the bifacially engineered devices show reproducible resistive switching with an *I*_on_/*I*_off_ ratio 3.67 × 10^5^, excellent endurance over 11 000 cycles, and retention time exceeding 10^5^ s, representing a state‐of‐the‐art of reliability and performance among the MHP‐based memristors for analog neuromorphic computing applications.^[^ ^41^ ^]^ The proposed devices display a high linearity factor during LTP and LTD, specifically *A*_P_ = 1.42 and *A*_D_ = 1.19, with a low symmetric error (*S*) of 0.07, which serves as a gauge parameter comparing the symmetricity among the reported MHP memristors.^[^ ^42^ ^]^ In terms of pragmatic applicability in neuromorphic computing, our bifacially engineered MHP memristors exhibit high accuracy of 92.60% and 94.53% according to the Canadian Institute for Advanced Research 10 (CIFAR‐10) dataset and Modified National Institute of Standards and Technology (MNIST) dataset, respectively, in a multilayer perceptron (MLP) simulation.^[^ ^43^, ^44^ ^]^ These results highlight the efficacy of the bifacial engineering strategy in overcoming the traditional limitations of MHP‐based memristors and advancing their applicability in neuromorphic systems. Consequently, our novel MHP configuration will make a significant advancement in the field of neuromorphic computing and artificial intelligence.

## 2 Results and Discussion

### 2.1 Overall Effects of Bifacial Engineering and Device Architectures

**Figure 1:**
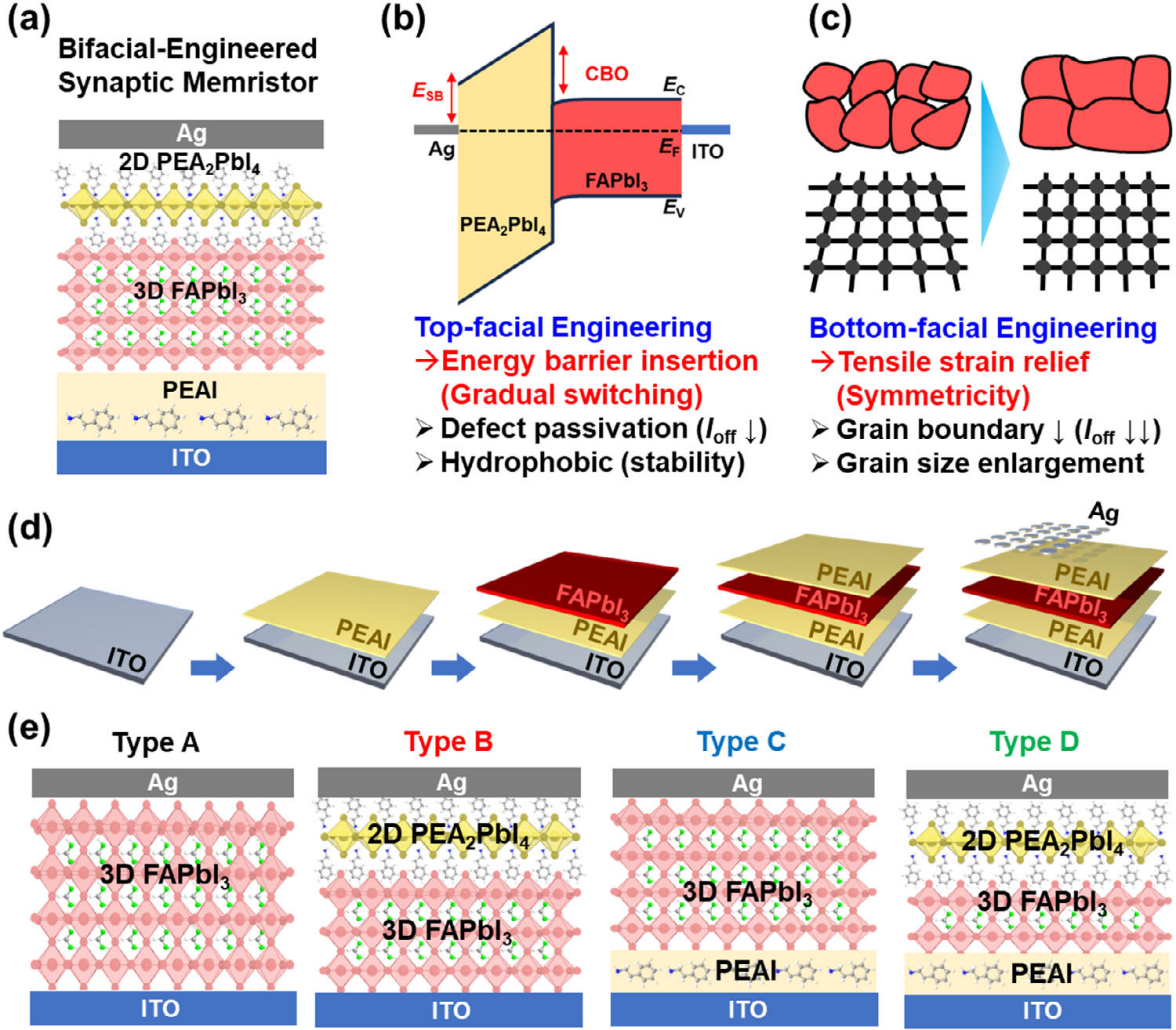
a) Schematic representation illustrating the bifacially engineered MHP memristor in this work. b) Energy band diagram highlighting top‐facial engineering for gradual energy barrier switching. c) Cartoon illustrating bottom‐facial engineering for symmetric tensile strain relief. d) Sequential fabrication step illustration of a bifacially engineered MHP memristor. e) Cross‐sectional configurations for memristor devices (Types A, B, C, and D), detailing the precise layering of each Type.

MHPs typically exhibit an ABX_3_ structure, in which “A” represents an organic or inorganic cation, “B” denotes a metal cation, and “X” signifies a halide anion. These materials can crystallize into different structural dimensions, namely 3D or 2D perovskites, depending on the choice of organic cations.^[^ ^45^ ^]^ We employed bifacial engineering by applying phenylethylammonium iodide (PEAI), an organic cation, to both the top and bottom surfaces of formamidinium lead iodide (FAPbI_3_). This strategy was designed to induce the formation of a 2D perovskite layer and alleviate tensile strain within the perovskite structure (**Figure** **1**a). Application of PEAI on the top surface of the perovskite layer successfully generated a hydrophobic 2D perovskite layer with reduced charge defects.^[^ ^22^, ^46^, ^47^, ^48^ ^]^ Consequently, this improved device stability and decreased the off‐current, *I*_off_ (Figure 1b). Additionally, the formation of a PEAI‐derived 2D layer elevated both the SB height and CBO, facilitating gradual switching behavior, thereby enhancing the LTP linearity of memristor operation and increasing the *I*_on_/*I*_off_ ratio. Interestingly, the PEAI treatment applied to the bottom surface did not yield a similar 2D perovskite formation. However, it reduced the number of voids and significantly increased the grain size of the perovskite layer,^[^ ^49^, ^50^ ^]^ as depicted in Figure 1c. This structural improvement facilitated the symmetricity, promoted LTD linearity, and further enhanced *I*_on_/*I*_off_ ratio and device reliability. To evaluate these effects systematically, MHP memristors were fabricated under varying device architectures: without the PEAI treatment, with only monofacial treatment (top or bottom), and with bifacial treatment for both top and bottom interfaces. Figure 1d outlines the fabrication sequence for these MHP memristors. Specifically, four configurations of MHP memristors were prepared (Figure 1e): glass/ITO/FAPbI_3_/Ag (Type A – no treatment); glass/ITO/FAPbI_3_/PEAI/Ag (Type B – top monofacial); glass/ITO/PEAI/FAPbI_3_/Ag (Type C – bottom monofacial); and glass/ITO/PEAI/FAPbI_3_/PEAI/Ag (Type D – bifacial). The corresponding experimental data and interpretations related to the different PEAI treatments are presented and discussed in detail in the following section.

### 2.2 Material Characterizations

**Figure 2:**
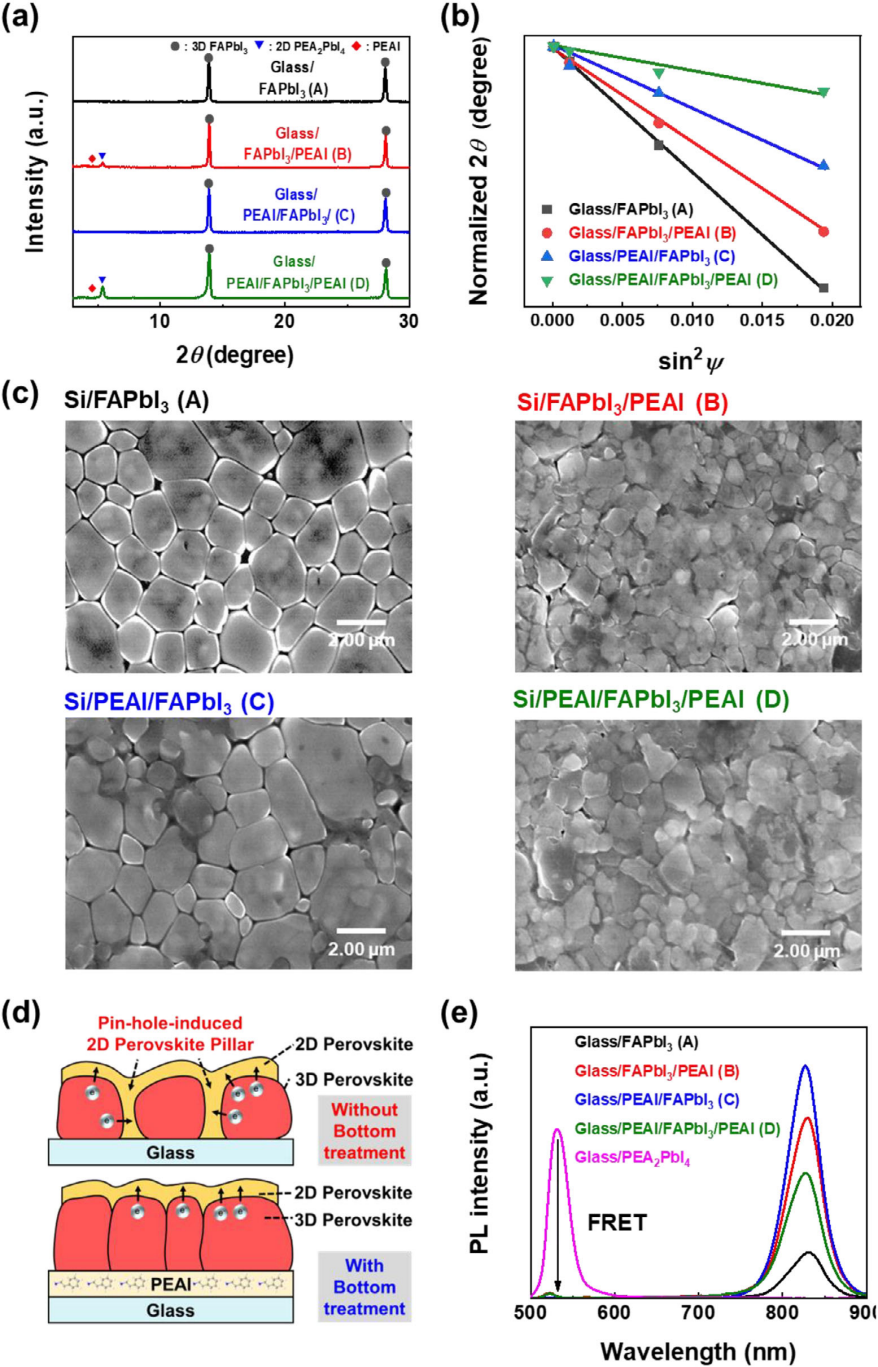
Structural, mechanical, morphological, and photophysical analyses of different perovskite film Types. a) X‐ray diffraction (XRD) patterns showing characteristic peaks corresponding to 3D FAPbI_3_ and 2D PEA_2_PbI_4_ in Types A–D. b) 2𝜃–sin^2^ *ψ* plot derived from the XRD data, illustrating tensile strain of Types A–D with respect to the incident angle *ψ*. c) SEM images comparing surface morphology changes induced by incorporating PEAI treatment for sample Types A–D. d) Schematic illustration comparing the crystallization process without (top) and with (bottom) PEAI bottom treatment. Bottom treatment suppresses pinhole formation and enhances grain growth by passivating the substrate interface. e) Steady‐state PL spectra of Types A–D and a reference 2D perovskite film (PEA_2_PbI_4_). The 2D and 3D perovskite layers exhibit characteristic PL peaks at ≈525 and 830 nm, respectively.

The properties of all four Types were analyzed. Grazing incidence X‐ray diffraction (GIXRD) measurements were performed to examine the formation of 2D perovskite phases and evaluate tensile strain within the MHP films across different configurations (Figures S1 and S2, Supporting Information). The absence of PbI_2_ and quasi‐2D PEA_2_PbI_4_ peaks confirms the high quality of the MHP films in all samples (**Figure** **2**a; Figure S1, Supporting Information).^[^ ^22^, ^51^, ^52^, ^53^ ^]^ At a grazing incidence angle (*ψ*) of 0.5°, a characteristic diffraction peak corresponding to the (002) plane of 2D PEA_2_PbI_4_ (2*θ* ≈ 5.4°) was observed exclusively in samples Type B and D, confirming the successful formation of 2D perovskites upon applying PEAI on top. Conversely, no formation of 2D perovskite was observed when PEAI was applied solely to the bottom surface (Figure 2a). It is well‐established that tensile strain in MHP films corresponds to elongated interplanar distances and increased defect density, predominantly near the substrate interface.^[^ ^49^, ^54^, ^55^ ^]^ Such strain‐induced defects typically arise from lattice distortions during crystallization, adversely impacting device stability and performance.^[^ ^49^, ^56^, ^57^ ^]^ The GIXRD analysis of the FAPbI_3_ (110) plane (2*θ* ≈ 14°) at varying incident angles (*ψ* = 0.5°, 2°, 5°, and 8°) reveals shifts toward lower 2*θ* values, indicative of strain‐induced lattice expansion (Figure S1, Supporting Information). To quantify residual tensile strain, the slope of the 2𝜃–sin^2^𝜑 relationship was examined (Figure 2b). Types C and D, with bottom PEAI layers, exhibited significantly reduced slope gradients, confirming reduced tensile strain and subsequently fewer defects compared to Types A and B without the bottom PEAI layers.^[^ ^49^, ^58^, ^59^ ^]^ The presence of PEAI on top also mitigated strain to some extent, suggesting optimal performance when bifacial PEAI coating is implemented, ensuring better symmetricity for memristor devices.^[^ ^60^ ^]^ The top‐view scanning electron microscopy (SEM) images of Type A–D were taken to examine the effect of tensile strain on the state of the perovskite grains (Figure 2c). It is evident that the voids in the perovskite films formed on glass (Type A) disappear for the MHP films on PEAI (Type C). In addition, Type C films exhibited less uniform grain sizes compared to Type A films, but the grain sizes became larger in the process of filling voids. The SEM images of Type B and Type D reveal 2D perovskites covering the 3D perovskite film. It can be inferred that in Type B, the 2D perovskites fill the voids by forming both vertical perovskite pillars and a horizontal film (Figure 2d).^[^ ^61^, ^62^ ^]^ Steady‐state photoluminescence (PL) spectra of Types A–D were acquired to investigate further, along with a pure 2D perovskite film as a reference for comparison (Figure 2e).^[^ ^63^, ^64^, ^65^ ^]^ Types B and D exhibited a PL peak at a wavelength (λ) of 525 nm (2D peak) corresponding to the bandgap of 2D PEA_2_PbI_4_, whereas Type C did not, confirming that the top PEAI coating induces the formation of 2D perovskites.^[^ ^46^ ^]^ As expected, all samples from Type A to D showed prominent PL peaks at approximately λ = 830 nm (3D peak), corresponding to 3D FAPbI_3_. Interestingly, Types C and D, which had bottom PEAI treatments, displayed a blueshift of ≈4 nm compared to Types A and B (Figure S3, Supporting Information).^[^ ^66^ ^]^ This observation supports the earlier assertion of reduced total defect density resulting from diminished tensile strain. The PL peak positions of the samples follow the gradient trend of tensile strain presented in Figure 2b. Bifacially engineered Type D is expected to have the lowest defect density, whereas Type A, with no PEAI treatment, is anticipated to have the highest defect density. To gain deeper insight into the recombination dynamics, time‐resolved photoluminescence (TRPL) measurements were performed (Figures S4 and S5 and Tables S1 and S2, Supporting Information). The intensity of TRPL decay curves was fit using a bi‐exponential model, *I(t)* = *A*_1_ exp(−*t*/*τ*_1_) + *A*_2_ exp(−*t*/*τ*_2_), where *τ*_1_ and *τ*_2_ represent non‐radiative (fast) and radiative (slow) recombination lifetimes, respectively. *A*_1_ and *A*_2_ denote their corresponding amplitudes.^[^ ^67^, ^68^, ^69^, ^70^ ^]^ In addition, a pulsed laser with an excitation wavelength (*λ*_ex_) of 372 nm was employed. TRPL decays were examined at an emission wavelength of 525 nm for Type B, Type D, and the reference 2D PEA_2_PbI_4_, which corresponds to the emission from the 2D perovskite layer (Figure S4 and Table S1, Supporting Information). The decay profiles of all three samples were clearly distinguishable from the instrument response function (IRF), indicating high temporal resolution. Both Type B and Type D exhibit a faster decay and shorter *τ*_1_ and *τ*_2_ compared to the 2D PEA_2_PbI_4_, which is attributed to Förster resonance energy transfer (FRET) from the 2D to the underlying 3D perovskite layer. This observation aligns well with the noticeably lower peak intensities of Type B and Type D relative to the pure 2D perovskite at the λ = 525 nm region (Figure 2e). Non‐radiative recombination pathways in both Type B and D, particularly FRET at the 2D/3D interface, are more dominant than radiative recombination.^[^ ^70^ ^]^ This non‐radiative mechanism competes with radiative pathways in the 3D perovskite, thus reducing the slow radiative recombination contribution. Normalizing the *A*_1_ and *A*_2_ values of the TRPL data for Type B, Type D, and the 2D perovskite reference, we were able to intuitively assess the relative contributions of different recombination pathways. The results clearly indicate that non‐radiative recombination is highly dominant in Type B, whereas Type D exhibits a comparable level of radiative recombination to that of the pure 2D perovskite. As previously discussed in Figure 2d, this difference can be ascribed to the distinct structural characteristics: while Type D contains only horizontal 2D perovskite layers, Type B likely forms vertical 2D perovskite pillars during the void passivation process, which facilitates multidirectional non‐radiative recombination pathways. Further, TRPL decay curves of Type A–D were measured at an emission wavelength of 830 nm (Figure S5 and Table S2, Supporting Information). Interestingly, τ_1_ for Type B and D is longer than that of A and C, indicating that the presence of the 2D layer introduces FRET processes that delay the early‐stage decay, possibly due to energy transfer dynamics across the 2D/3D interface. However, *τ*_2_ is shorter for Type B and D than for Type A and C due to the presence of non‐radiative recombination process.^[^ ^70^ ^]^ When comparing the TRPL lifetime of Type B and D, *τ*_1_ of Type D is slightly longer than that of Type B, which is attributed not only to more controlled FRET from the 2D to 3D perovskite layer, but also to a lower density of the 2D/3D interfacial defects. In contrast, the shorter τ_1_ in Type B is associated with enhanced non‐radiative recombination caused by vertical 2D perovskite pillars and a higher density of interfacial defects (Figure 2d). Similarly, *τ*_2_ of Type D is substantially longer than that of Type B, owing to its more uniform interface and horizontal 2D perovskite layer enabled by the bifacial PEAI treatment. On the contrary, the shorter *τ*_2_ observed in Type B reflects the compounded effect of severe defect‐assisted recombination and multidirectional carrier loss facilitated by the vertical 2D perovskite pillar. Therefore, the TRPL analysis is consistent with the structural and optical characteristics previously discussed in Figure 2d,e. Although TRPL results indicate that Type D exhibits suppressed non‐radiative recombination due to improved film quality enabled by bottom PEAI treatment, its steady‐state PL intensity is lower than that of Type B. This discrepancy is attributed to stronger optical screening caused by a thicker and more continuous 2D perovskite layer in Type D than that of Type B (Figure 2e; Figure S6, Supporting Information). Thus, Type C showed the highest 3D PL intensity, as it had lower defect density and reduced non‐radiative recombination due to the bottom PEAI treatment (Figure 2e). The absence of a 2D perovskite layer in Type C results in no optical screening, further contributing to the strong 3D PL emission. This was followed by Types B, D, and A, in descending order. The lowest PL intensity at ≈830 nm was observed in Type A, attributed to the presence of voids. The Fourier transform infrared (FTIR) analysis confirms the previous findings, as the peak shifts of PEAI and FAI components in the perovskite indicate the degree of their interactions (Figure S7, Supporting Information). The absence of PEAI bond stretching in Type C suggests that the FTIR signal reflects interactions involving only the top PEAI layer (Figure S8, Supporting Information). Especially, the C–N stretching mode of PEAI exhibits a substantial redshift, indicating strong interactions with the perovskite. Similarly, shifts in the N‐H and C = N stretching modes of the perovskite are observed, particularly in Type B and Type D, providing clear evidence for the formation of 2D perovskite layers on the surface (Figure S9, Supporting Information). Furthermore, UV–vis absorption spectra and the corresponding Tauc plots (Figure S10, Supporting Information) confirm that all Types A–D possess identical bandgaps, supporting the conclusions drawn from the steady‐state PL and TRPL analyses.

### 2.3 Device Characterizations

**Figure 3:**
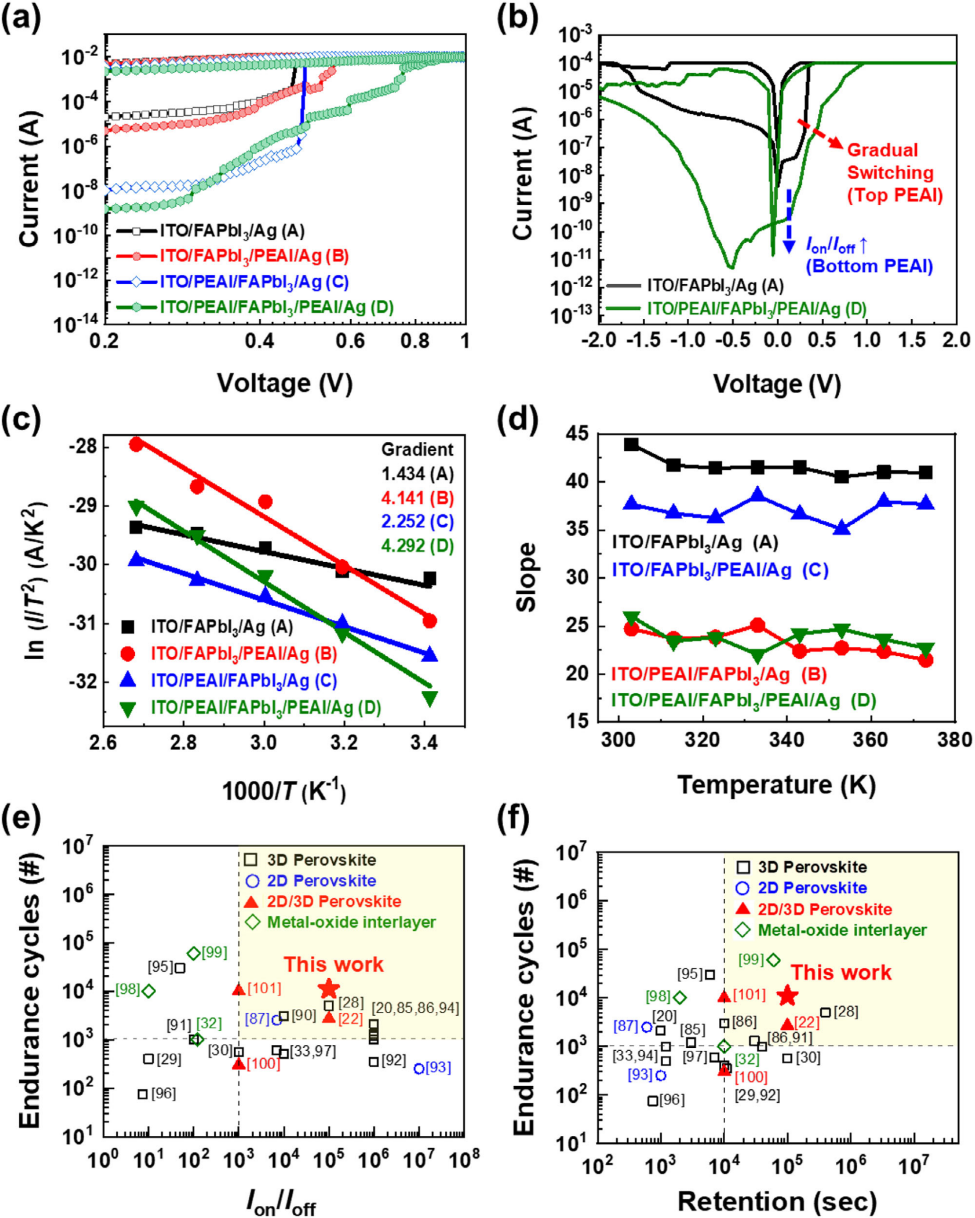
a) Log *I* – log *V* sweep curves of four different device structures: Type A–ITO/FAPbI_3_/Ag, Type B– ITO/FAPbI_3_/PEAI/Ag, Type C– ITO/PEAI/FAPbI_3_/Ag, and Type D– ITO/PEAI/FAPbI_3_/PEAI/Ag. b) Enlarged view of the *I–V* graphs of Type A and D, highlighting the gradual switching and enhanced *I*_on_/*I*_off_ ratio owing to lowered *I*_off_ in Type D (indicated by red arrows). c) ln (*I*/*T* ^2^) versus 1000/*T* plot of all four devices extracted from the low‐voltage regime, with calculated gradients corresponding to each device, indicating variations in the SB heights. The gradients for Types A, B, C, and D are 1.434, 4.141, 2.252, and 4.292, respectively. d) Extracted slope versus *T* graph of all four devices, where slope is calculated from ln (*I*) versus *V* ^0.5^ plot (Figure S17, Supporting Information). The extracted slope means SB thickness of the synaptic memristors. Record plots benchmarking of the performance (*I*_on_/*I*_off_) and reliability (endurance and retention time) of perovskite memristors for e) endurance cycles versus *I*_on_/*I*_off_ and for f) endurance cycles versus retention time; devices are categorized as 3D perovskite (black square),^[^ ^20^, ^28^, ^29^, ^30^, ^33^, ^85^ ^,^ ^86^, ^90^, ^91^, ^92^, ^94^, ^95^, ^96^, ^97^ ^]^ 2D perovskite (blue circle),^[^ ^87^, ^93^ ^]^ 2D/3D perovskite (red triangle),^[^ ^22^, ^100^, ^101^ ^]^ and metal‐oxide interlayers (green diamond).^[^ ^32^, ^98^, ^99^ ^]^ Our device (“This work,” shown as a red star) displays an excellent balance of endurance cycle, *I*_on_/*I*_off_, and retention time.

The electrical characteristics of the four device configurations were thoroughly assessed and comparatively analyzed. Specifically, *I*_on_ and *I*_off_, corresponding to the low‐ and high‐resistance (*R*) state, respectively, were determined at a fixed read voltage of 0.1 V. The *I–V* graphs of all four Type devices showing nonvolatile bipolar switching characteristics with counter‐clockwise direction are depicted in Figure S11 (Supporting Information), and *I*_on_, *I*_off_, and *I*_on_/*I*_off_ ratio are summarized in Table S3 (Supporting Information).^[^ ^30^, ^71^ ^]^ It is worth noting that the PEAI concentration used for the fabrication is optimized to be 20 mg/mL, which gave the highest *I*_on_/*I*_off_ ratio (Figure S12, Supporting Information). To elucidate the intrinsic switching mechanism of our memristors, the correlation between *I*_on_, *I*_off_, and device area was explored systematically (Figure S13, Supporting Information).^[^ ^32^, ^72^, ^73^, ^74^ ^]^ It was observed that *I*_on_ exhibited mild dependence on device area, whereas *I*_off_ more markedly decreased with scaling down the device area. This phenomenon is indicative of a CF‐based switching mechanism predominantly governed by electrochemical metallization (ECM).^[^ ^73^ ^]^ In addition, we examined the temperature‐dependent *R* characteristics in both the on‐ and off‐state of our devices to clearly support the ECM‐based filamentary mechanism in our devices (Figure S14, Supporting Information).^[^ ^75^, ^76^, ^77^ ^]^ From Figure S14 (Supporting Information), we extracted the temperature coefficient of *R* (TCR), defined as (1/*R*_0_) × (*dR*/*dT*), where *R*_0_ is the *R* at the room temperature and *T* is the absolute temperature. The positive TCR in the on‐state and the negative TCR in the off‐state provide compelling evidence for the formation of metallic conductive filaments and semiconducting behavior, respectively.^[^ ^77^ ^]^ Logarithmic current‐voltage (log *I*–log *V*) analyses revealed significantly lower *I*_off_ values and enhanced *I*_on_/*I*_off_ ratios for device Types C and D compared to Types A and B (**Figure** **3**a; Table S3, Supporting Information). This results primarily from reduced defect densities and mitigation of structural voids within MHP films achieved through bottom PEAI treatment as discussed in Figure 2. Furthermore, Types B and D manifest a gradual switching behavior, which is in contrast to the abrupt switching characteristic observed in Types A and C (Figure 3a; Figure S11, Supporting Information).^[^ ^32^, ^78^ ^]^ According to the ultraviolet photoelectron spectroscopy (UPS) and ultraviolet–visible (UV–vis) spectroscopy measurements, it is confirmed that the obvious CBO is formed at 2D/3D interface (Figures S15 and S16, Supporting Information).^[^ ^79^, ^80^, ^81^ ^]^ It means that the top PEAI treatment increases the CBO introduced by the 2D perovskite layer, effectively suppressing CF formation.^[^ ^6^, ^32^ ^]^ In addition, it is noteworthy that *I*_off_ of Type B and Type D are lower than those of Type A and Type C, respectively, due to the defect passivation by the 2D perovskites. Overall, the bifacially engineered Type D device demonstrated the best performance. Comparative *I–V* graphs of Type A and Type D devices affirmed the presence of bipolar switching, which is a crucial property for nonvolatile synaptic memristors (Figure 3b). It should be noted that Type D showed substantially reduced *I*_off_ owing to the alleviation of tensile strain via bottom PEAI treatment as well as a gradual switching behavior attributed to the CBO introduced by the top PEAI treatment. To further investigate the origin of the gradual switching behavior observed in Types B and D, resulting from the formation of 2D perovskites, SB height and thickness were empirically extracted from temperature‐dependent *I–V* characteristics (Figure S17, Supporting Information) using the Richardson‐Schottky Equation (1):

(1) $$I=AT^{2}\exp\left[\frac{-q\phi_{B}-\sqrt{qV/\left(4\pi \varepsilon_{r}\varepsilon_{0}d\right)}}{k_{B}T}\right]$$

where *A* represents the Richardson constant, *T* is the absolute temperature, *q* denotes the electron charge, *k*_B_ is the Boltzmann constant, *qϕ*_B_ signifies the SB height, *ε*_r_ is the optical dielectric constant, *ε*_0_ is the permittivity in a vacuum, and *d* is the SB thickness.^[^ ^20^, ^21^, ^82^, ^83^ ^]^ Graphs of ln (*I*/*T* ^2^)–1000/*T* were analyzed, derived from the temperature‐dependent *I–V* characteristics at around *I*_off_ region (Figure 3c). The linear behavior observed in all four devices confirms that the Schottky emission mechanism governs charge transport. The gradient of each curve corresponds to the SB height, with extracted values of 1.434, 4.141, 2.252, and 4.292 for the Type A, B, C, and D, respectively. The slopes of Types B and D are much higher than those of Types A and C, revealing that Types B and D exhibit higher SB heights. Of particular note, the significantly elevated barrier heights in Types B and D are attributed to the 2D perovskite layer at the top interface, which increases the SB height between the nonmetallic 3D perovskite and the metal electrode. A high CBO serves as a prerequisite for gradual switching by suppressing abrupt CF growth, which explains the behavior observed in Types B and D.^[^ ^32^, ^78^ ^]^ Correspondingly, the ln (*I*/*T* ^2^) values of the Types A–D measured at the 293 K (1000/*T* ≈3.4) aligned well with previously observed *I*_off_ trends as shown in Figure 3a. It is inferred that the presence of 2D perovskite can also contribute to lowering the *I*_off_, while the reduced tensile strain due to the PEAI bottom treatment is the main contributor. The temperature dependent ln *I* versus *V* ^0.5^ graphs at the switching region provide valuable insights into the *d* (Figure 3d; Figure S18, Supporting Information). The observed linear relationship in all device types further confirms that the dominant conduction mechanism is governed by Schottky emission. According to the Equation (1), *d* is inversely proportional to the square of the slope, i.e., *d* ∝ (1/slope)^2^.^[^ ^20^ ^]^ The calculated slopes for Types A and C are ≈42 and 38, respectively, while those for Types B and D are similar at around 25 (Figure 3d; Figure S18, Supporting Information). The remarkably lower slopes of Types B and D compared to Types A and C indicate the formation of a thicker SB, which verifies the contribution of the 2D perovskite layers.^[^ ^20^ ^]^ Similarly, the slopes of the four devices were extracted in the vicinity of *I*_off_ region, which is a commonly analyzed regime for evaluating Schottky emission. The extracted slope for Type A and C is ≈0.6 and 0.8, while those of Type B and D exhibited similar values around 0.3, respectively (Figure S17e, Supporting Information). These results strongly suggest that the *d* in Types B and D is significantly larger than in Types A and C, supporting the successful formation of 2D perovskites in Types B and D. It is worth noting that the slope of Type C is slightly higher than that of Type A, which can be attributed to the higher density of defects in Type A. These defects lead to enhanced electric field screening, thereby suppressing Schottky emission and reducing the apparent slope.^[^ ^21^, ^84^ ^]^ This interpretation is further supported by a complementary yet simpler Arrhenius analysis using the activation energies extracted from Equation (2) (Figures S19 and S20, Supporting Information),^[^ ^21^ ^]^

(2) $$\ln\left(I_{off}\right)=\ln\left(I_{0}\right)-\frac{E_{a}}{k_{B}T}$$

where *I*_o_ is a constant, *E*_a_ represents the activation energy. The UPS and UV–vis measurements were further performed to corroborate these findings and visualize the corresponding energy band diagrams, which show excellent agreement with our hypothesis (Figures S15 and S16, Supporting Information).^[^ ^79^, ^80^, ^81^ ^]^

The bifacially engineered Type D device, which exhibited the best performance in terms of reliability, was subjected to endurance and retention tests. Figure S21a (Supporting Information) presents the endurance characteristics of the device, sustaining over 11000 direct‐current (DC) switching cycles while maintaining an *I*_on_/*I*_off_ ratio exceeding 10^5^, which is comparable to the best reported values.^[^ ^28^, ^85^, ^86^ ^]^ In addition, Figure S21b (Supporting Information) shows that the device demonstrated excellent retention characteristics, maintaining stable performance for over 10^5^ s, thereby fulfilling the essential requirements of nonvolatile synaptic devices.^[^ ^22^, ^28^, ^30^ ^]^ It is worth noting that DC stress conditions are generally considered more severe than pulse stress conditions.^[^ ^87^ ^]^ Furthermore, the performance parameters, including *I*_on_/*I*_off_ ratio, endurance, and retention time, are benchmarked and summarized in Figure 3e,f and **Table** **1**.^[^ ^20^, ^22^, ^28^, ^29^, ^30^, ^32^, ^33^, ^85^, ^86^, ^87^, ^90^, ^91^, ^92^, ^93^, ^94^, ^95^, ^96^, ^97^, ^98^, ^99^, ^100^, ^101^ ^]^ As it is critical to simultaneously satisfy both the performance and reliability of memristors, we defined a performance volume metric, calculated as the product of *I*_on_/*I*_off_ ratio, endurance, and retention time. Overall, our device demonstrates the highest performance volume reported to date among MHP‐based memristors. Meanwhile, it has been widely reported that MHP‐based memristors suffer from poor endurance, with most studies achieving values ≈1000 cycles, underscoring the particular difficulty of enhancing endurance in pure MHP devices. To address this limitation, several works have introduced metal‐oxide interlayers, and Poddar et al., demonstrated exceptional endurance and retention characteristics.^[^ ^73^ ^]^ However, such improvements cannot be solely attributed to enhancements in the intrinsic performance of pure MHP materials.^[^ ^41^ ^]^ In this context, our Type D memristor not only surpasses the previously reported record‐high endurance of 5655 cycles for pure MHP‐based memristors but also simultaneously achieves high *I*_on_/*I*_off_ ratios and excellent retention characteristics.^[^ ^30^, ^88^ ^]^ Given the intrinsic moisture sensitivity of perovskite films, Type B and D devices with top‐surface PEAI passivation exhibited significantly improved morphological stability over 269 h (≈11 days) under high‐humidity conditions, compared to unpassivated devices (Figure S22, Supporting Information). These results clearly demonstrate the effectiveness of PEAI passivation in enhancing the environmental stability of perovskite‐based devices.^[^ ^48^, ^89^ ^]^

**Table 1:** Comparative benchmarks of the performance (*I*_on_/*I*_off_) and reliability (endurance and retention time) for reported MHP‐based memristors.

Structure	Type	*I*_on_/*I*_off_	Endurance (cycles)	Retention time [s]	Performance Volume (a.u.)	Year
ITO/PEAI/FAPbI_3_/PEAI/Ag	2D/3D perovskite	3.7 × 10^5^	1.1 × 10^4^	10^5^	4.07 × 10^14^	2025 (This work)
ITO/PEDOT:PSS/ CH_3_NH_3_PbI_3_/Cu	3D perovskite	10^4^	3.0 × 10^3^	10^4^	3 × 10^11^	2015^[^ ^90^ ^]^
PET/ITO/CH_3_NH_3_PbI_3_/ Au	3D perovskite	10	4 × 10^2^	10^4^	4 × 10^7^	2016^[^ ^29^ ^]^
FTO/CH_3_NH_3_PbI_3‐x_Cl_x_/Ag	3D perovskite	10^2^	10^3^	4.0 × 10^4^	4 × 10^9^	2016^[^ ^91^ ^]^
Si/SiO_2_/Ti/Pt/MAPbI_3_/Ag	3D perovskite	10^6^	3.5 × 10^2^	1.1 × 10^4^	3.85 × 10^12^	2016^[^ ^92^ ^]^
Si/SiO_2_/Ti/Pt/MAPbI_3_/Ag	3D perovskite	10^6^	1.3 × 10^3^	3.0 × 10^4^	3.9 × 10^13^	2017^[^ ^86^ ^]^
Si/SiO_2_/Ti/Pt/BA_2_Ma_n‐1_ Pb_n_I_3n+1_/Ag (n = 1, 2, …)	2D perovskite	10^7^	2.5 × 10^2^	1.0 × 10^3^	2.5 × 10^12^	2017^[^ ^93^ ^]^
Si/SiO_2_/Ti/Au/ CH_3_NH_3_PbI_3_/Au	3D perovskite	10^6^	10^3^	1.2 × 10^3^	1.2 × 10^12^	2017^[^ ^94^ ^]^
FTO/MAPbI_3‐x_Cl_x_/Ag	3D perovskite	50	3.0 × 10^4^	6.0 × 10^3^	9 × 10^9^	2017^[^ ^95^ ^]^
Ag/MAPbI_3_/Ag	3D perovskite	7.5	7.5 × 10^1^	7.5 × 10^2^	4.2 × 10^5^	2018^[^ ^96^ ^]^
Si/Pt/*δ*‐FAPb_3_I/Ag	3D perovskite	10^6^	1.2 × 10^3^	3.0 × 10^3^	3.6 × 10^12^	2018^[^ ^85^ ^]^
PET/ITO/PMMA/ CsPbBr_3_(QDs)/PMMA/ Ag	3D perovskite	10^5^	5.0 × 10^3^	4 × 10^5^	2 × 10^14^	2018^[^ ^28^ ^]^
ITO/MAPbI_3_/Ag	3D perovskite	10^4^	5.0 × 10^2^	1.2 × 10^3^	6 × 10^9^	2019^[^ ^33^ ^]^
Si/SiO_2_/Ti/Pt/CsSnI_3_/ PMMA/Ag	3D perovskite	7.0 × 10^3^	6.0 × 10^2^	7.0 × 10^3^	2.94 × 10^10^	2019^[^ ^97^ ^]^
FTO/CH_3_NH_3_PbI_3‐x_Cl_x_/Ag (epoxy tip)	Metal‐oxide interlayer	≈10	10^4^	2.0 × 10^3^	2 × 10^8^	2019^[^ ^98^ ^]^
Pt/BA_2_PbBr_4_/AgZnO/Al	Metal‐oxide interlayer	10^2^	6 × 10^4^	6 × 10^4^	3.6 × 10^11^	2020^[^ ^99^ ^]^
Pt/Ti/MAPbI_3_/PEAI/ PMMA/Ag	2D/3D perovskite	10^5^	2.7 × 10^3^	10^5^	2.7 × 10^13^	2020^[^ ^22^ ^]^
ITO/MAPbI_3‐x_Cl_x_/BAI/Al	2D/3D perovskite	10^3^	3 × 10^2^	10^4^	3 × 10^9^	2020^[^ ^100^ ^]^
ITO/PEDOT:PSS/pTPD/CsPbBr_3_/Ag	3D perovskite	10^3^	5.6 × 10^2^	1.0 × 10^5^	5.6 × 10^10^	2022^[^ ^30^ ^]^
ITO/PEA_2_MA_4_Pb_5_I_16_/Ag	2D perovskite	7 × 10^3^	2.5 × 10^3^	6.0 × 10^2^	1.05 × 10^10^	2023^[^ ^87^ ^]^
Pt/FAPbI_3_/PMMA/Ag	3D perovskite	10^6^	2.1 × 10^3^	10^3^	2.1 × 10^12^	2023^[^ ^20^ ^]^
ITO/CH_3_NH_3_PbI_3‐x_Cl_x_/ PEASCN/Al	2D/3D perovskite	10^3^	10^4^	10^4^	1 × 10^11^	2023^[^ ^101^ ^]^
ITO/SnO_2_/δ‐FAPbI_3_/Ag	Metal‐oxide interlayer	1.2 × 10^2^	10^3^	10^4^	1.2 × 10^9^	2024^[^ ^32^ ^]^

### 2.4 Applicability Evaluation of Neuromorphic Computing

**Figure 4:**
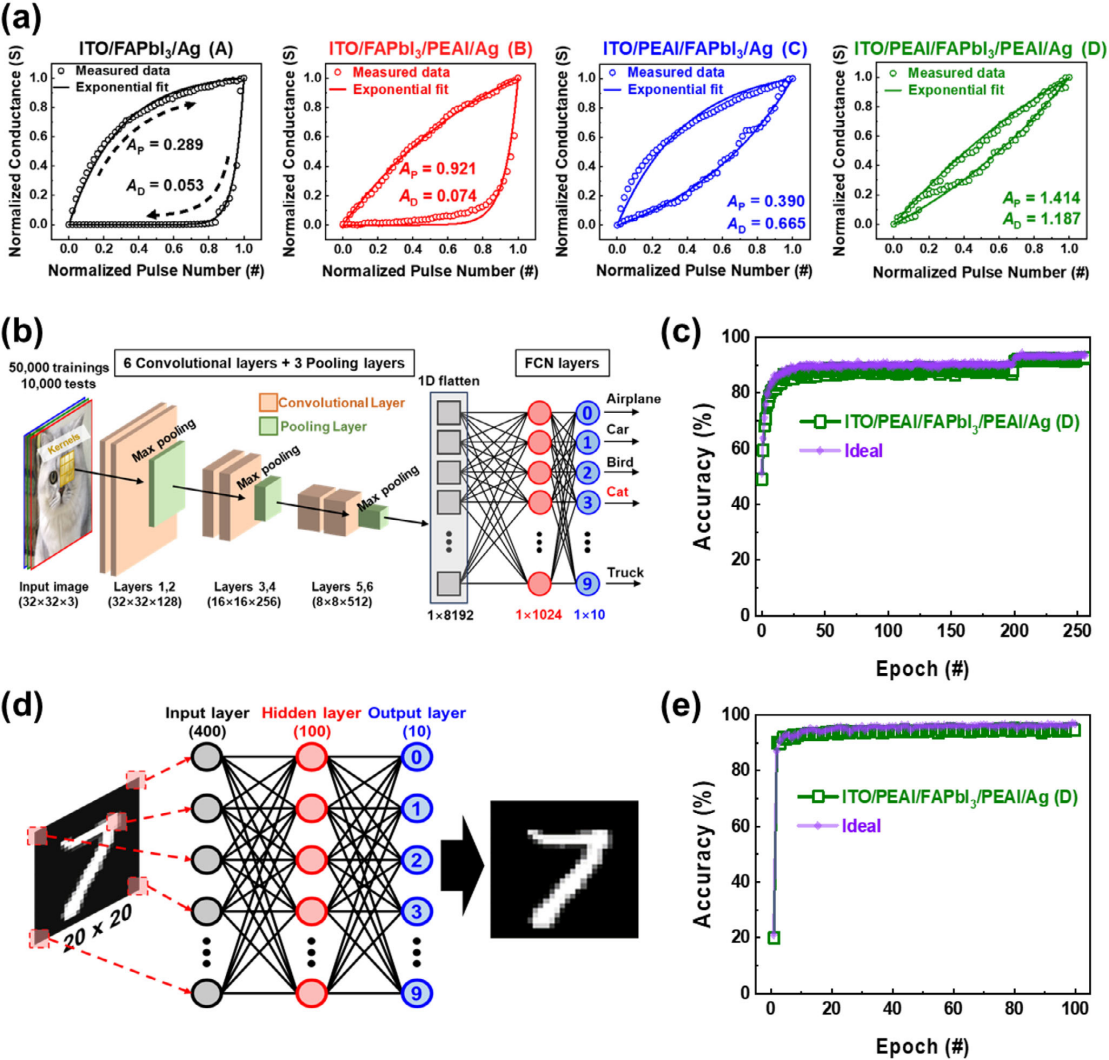
a) Normalized LTP and LTD curves of Types A–D during synaptic weight updates as a function of pulse number, with both experimental data and the corresponding exponential fitting results plotted together. Normalization for all Types A–D was carried out using (*G* – *G*_min_)/(*G*_max_ – *G*_min_). LTP and LTD were induced by applying 50 successive positive (0.6 V, 100 ms) and negative pulses (−0.6 V, 100 ms), respectively. b) CIFAR‐10 classification simulations employed a CNN based on the VGG‐8 architecture. c) Simulated recognition accuracies for the CIFAR‐10 dataset using Type D (92.60%) and the ideal synapse (93.52%) assumed in this work. d) Conceptual schematic of a handwritten MNIST digit pattern and the MLP architecture used for classification. e) Simulated recognition accuracies for the MNIST dataset using Type D (94.53%) and the ideal synapse (96.58%).

Having observed the record‐high performance volume of the bifacially engineered Type D device among the reported MHP‐based memristors, we tested synaptic plasticity, i.e., LTP and LTD curves of the same device during the synaptic weight update (*W* update) for the purpose of evaluating high linearity and symmetricity. Generally, synaptic plasticity, which is one of the most important characteristics of the nonvolatile memristors, is measured as a conductance (*G*) modulation with respect to the applied pulse number.^[^ ^102^, ^103^ ^]^ Synaptic memristors should exhibit clearly distinguishable *G* states as a function of the applied pulse number to ensure reliable *W* updates.^[^ ^30^, ^103^ ^]^ Moreover, it is desirable for the synaptic memristors to exhibit linear *G* modulation in both LTP and LTD processes. To demonstrate the synaptic plasticity of the Types A–D, positive and negative voltage pulse trains (0.6 V, 100 ms) and (−0.6 V, 100 ms) were applied for LTP and LTD processes, respectively (Figure S23, Supporting Information). 50 pulses were applied for each of the LTP and LTD processes, and the corresponding cycle‐to‐cycle measurement results are shown in Figure S24 (Supporting Information). To evaluate the linearity of *G* modulation in Types A–D, an exponential fitting model was employed. This model underpins the hardware‐based MLP simulator used for CIFAR‐10^[^ ^104^ ^]^ and MNIST classification.^[^ ^43^, ^44^ ^]^ As shown in Equations (3)–(5), this model was independently applied to the *G* during LTP and LTD processes to extract the fitting parameters *A*_P_ and *A*_D_, which represent the linearity factors associated with potentiation and depression, respectively.

(3) $$G_{LTP}=B_{P}\left(1-\exp\left(-\frac{P}{A_{P}}\right)+G_{\min}\right.$$

(4) $$G_{LTD}=-B_{D}\left(1-\exp\left(\frac{P-P_{\max}}{A_{D}}\right)+G_{\max}\right.$$

(5) $$B_{P,D}=\left(G_{\max}-G_{\min}\right)\left(1-\exp\left(-\frac{P_{\max}}{A_{P,D}}\right)\right)$$

where *G*_LTP_ and *G*_LTD_ represent *G* during the LTP and LTD, while *G*_max_ and *G*_min_ denote the maximum and minimum *G* of the LTP and LTD, respectively. *P* and *P*_max_ correspond to the pulse number associated with the *G* and the total number of applied pulses, respectively. *B*_P_ and *B*_D_ are scaling parameters used to fit the LTP and LTD curves, respectively. It should be emphasized that high values of both *A*_P_ and *A*_D_ are required in high performance synaptic memristors. The exponential fitting results of all four devices after normalization, i.e., (*G* – *G*_min_)/(*G*_max_ – *G*_min_) are presented in **Figure** **4**a, and the unprocessed LTP and LTD curves before fitting are shown in Figure S23 (Supporting Information).^[^ ^6^, ^44^, ^105^ ^]^ In Figure 4a, Type A shows lowest *A*_P_ and *A*_D_. Meanwhile, Type B exhibited a higher *A*_P_ than those of Types A and C, indicating the effectiveness of the top PEAI treatment. This enhancement is attributed to the elevated CBO induced by the 2D perovskite layer, which enables precise control over the CF formation. However, the *A*_D_ value of Type B was similar to that of Type A, suggesting that the top PEAI treatment has a limited impact on the LTD process. In contrast, Type C, which underwent bottom PEAI treatment, exhibited a significantly higher *A*_D_ than Types A and B. This improvement is attributed to the reduction in grain boundaries and defects due to the relief of tensile strain thanks to the bottom PEAI treatment, enabling controlled CF rupture. Since most synaptic devices exhibit greater difficulty in achieving high linearity during the rupture process compared to the forming process, improving *A*_D_ is particularly important.^[^ ^7^ ^]^ It should be highlighted that the slightly higher *A*_P_ of Type C compared to Type A suggests that improved MHP film quality also contributes to enhancing *A*_P_ as well as *A*_D_. Consequently, the bifacially engineered Type D device exhibited the highest *A*_P_ and *A*_D_ in the LTP and LTD processes, respectively, indicative of a synergistic effect of the bifacial PEAI treatment without the need for oxide layers. These results collectively demonstrate that the bifacial PEAI treatments primarily affect the CF formation and rupture processes as reflected in the synaptic memristor characteristics. To further support the above discussion, the CF formation and rupture processes in Types B and D are illustrated with the energy band diagrams (Figure S25, Supporting Information). *S* was defined to evaluate the symmetricity as shown in Figure S26 (Supporting Information). The calculated *S* values for Types A–D were 0.673, 0.444, 0.113, and 0.070, respectively, where low values indicating high symmetricity.^[^ ^103^ ^]^ From these results, we can see that symmetricity is significantly enhanced by the bottom PEAI treatment, as demonstrated by the reduced *S* value of Type C relative to Types A and B. It reveals that the symmetricity of MHP‐based synaptic memristors is improved by reducing the defects and tensile strain. Type D shows the lowest *S* value, which also supports the synergistic effect of bifacial PEAI treatment in enhancing the MHP‐based synaptic memristors. Incidentally, the calculated *S* value of our Type D is superior to the previously reported other synaptic memristors.^[^ ^103^ ^]^ The *A*_P_ and *A*_D_ values, along with their associated nonlinearity indices *α*_P_ and *α*_D_, as well as *S* and *G*_max_/*G*_min_ are summarized in Table S4 (Supporting Information).

To evaluate and demonstrate the applicability of the bifacially engineered Type D as a synaptic memristor, we conducted CIFAR‐10 classification simulations. In this simulation, we employed a convolutional neural network (CNN) based on the visual geometry group (VGG)‐8 architecture, implemented within the hardware‐aware deep neural network (DNN) framework of the DNN+NeuroSim v2.1 simulation platform.^[^ ^104^, ^106^ ^]^ Further, we benchmarked the offline training method in this simulation, meaning that the *W* update does not occur during the inference operation. Our neural network architecture consists of six convolutional layers, three max‐pooling layers, and two fully‐connected neuron (FCN) layers as depicted in Figure 4b. Specifically, it processes 50 000 training and 10 000 test samples, each comprising 32 × 32 × 3 image inputs representing the red, green, and blue color channels. These inputs are sequentially passed through three convolutional blocks, each containing two convolutional layers followed by a max‐pooling layer, with 128, 256, and 512 kernels, respectively. After the convolutional and pooling operations, the resulting feature maps are flattened into a 1D vector of length 8192, which is fed into a FCN layer with 1024 neurons. This is followed by a final FCN layer producing 10 outputs corresponding to the CIFAR‐10 classes, from which a predicted class is selected. Feed‐forward and back propagation are sequentially performed for inference and training, respectively. The detailed description of feed‐forward and back propagation for the simulation is explained in Note S1 (Supporting Information). Consequently, we achieved a high accuracy of 92.60% at epoch 256, and this is owing to the high linearity of the Type D device (Figure 4c). Besides, the CIFAR‐10 simulation result of our Type D is compared with the previous reports of MHP‐based synaptic memristors in Table S5 (Supporting Information), showing the highest accuracy because of the high linearity.^[^ ^6^, ^23^, ^31^, ^32^, ^33^, ^34^, ^35^, ^42^, ^108^, ^109^, ^110^, ^111^, ^112^ ^]^ Additionally, it is comparable to the result of ideal synapses supposed in this simulation. The detailed parameters of ideal synapses are summarized in Table S4 (Supporting Information). To clarify the significance of high‐linearity synapses in the CIFAR‐10 simulation, the accuracy is mainly determined by the linearity of the synaptic devices, which form the synaptic arrays (hidden layers) of the FCN layers. It should be noted that the *W* in FCN layers is defined as the difference between the *G* values of two equivalent Type D synapses, i.e., *W* = *G*_LTP_ – *G*_LTD_. To potentiate the *W* value, *G* of the Type D device corresponding to *G*_LTP_ is increased, while that of *G*_LTP_ is decreased (*W*↑ = *G*_LTP_↑ − *G*_LTD_↓). Conversely, to depress the *W*, the *G* value of *G*_LTP_ is decreased, and that of *G*_LTD_ is increased (*W*↓ = *G*_LTP_↓ − *G*_LTD_↑).^[^ ^103^, ^106^ ^]^ Therefore, backpropagation for updating the *W* to minimize the error during the feed‐forward propagation is effectively achieved when considering the high *A*_P_ and *A*_D_ values of Type D memristor, which correspond to the low nonlinearity indices *α*_P_ and *α*_D_ (Note S1, Supporting Information). Furthermore, we evaluated the feasibility of Type D through the MNIST pattern recognition test based on the MLP, which is one of the most widely adopted artificial neural networks for classification.^[^ ^107^ ^]^ MLP+NeuroSim v3.0 simulator was employed to reflect the synaptic behaviors of our devices, including the LTP and LTD characteristics.^[^ ^43^, ^44^ ^]^ Figure 4d presents a conceptual schematic illustrating the handwritten digit pattern with 20 × 20 pixel image for training or inference, together with the MLP. Moreover, the MLP architecture consists of three neuron layers: an input layer with 400 neurons, a hidden layer with 100 neurons, and an output layer with 10 neurons. The recognition accuracy was evaluated using the MNIST dataset over 100 epochs. A total of 60 000 and 10 000 distinct patterns were used for training and inference, respectively, with 8000 images utilized per epoch during training. Notably, *W* update mechanism employed in the FCN layers of the MNIST simulation is identical to that used in the FCN layers of the CIFAR‐10 simulation. As shown in Figure 4e, the simulation results reveal that the Type D synapse achieved 94.53%, while the ideal synapse assumed in this work reached an accuracy of 96.58%. These results were benchmarked against previously reported other MHP‐based synaptic memristors as summarized in Table S5 (Supporting Information). Interestingly, Type D clearly demonstrates the superior performance of our bifacial PEAI treatment strategy in enhancing MHP‐based synaptic devices, particularly in terms of pattern recognition accuracy and linear synaptic behavior. Furthermore, it highlights the strong potential of our proposed device platform for hardware implementation of neuromorphic networks, thereby opening new avenues for next‐generation MHP‐based neuromorphic computing technologies.

## 3 Conclusion

In this work, we demonstrated bifacially engineered MHP‐based synaptic memristors that simultaneously achieve high linearity, symmetricity, and reliability—key requirements for hardware implementation of neuromorphic computing. By introducing PEAI layers both above and below the FAPbI_3_ active layer, 2D/3D perovskite heterostructure with reduced defect and tensile strain was formed that effectively regulated CF formation and rupture processes. To be more specific, the top PEAI treatment facilitated the formation of a 2D PEA_2_PbI_4_ passivation layer, resulting in a CBO and elevated SB, which in turn enabled gradual switching behavior and suppressed Ag ion migration. Meanwhile, the bottom PEAI treatment relieved interfacial tensile strain and improved perovskite grain morphology, thereby enhancing filament uniformity and switching symmetry. The resulting bifacially engineered device exhibited a high *I*_on_/*I*_off_ ratio of 3.67 × 10^5^, stable endurance over 11 000 cycles, and retention time exceeding 10^5^ s—representing state‐of‐the‐art performance and reliability among the MHP‐based memristors to date. Furthermore, the synaptic characteristics of our device exhibited high linearity, specifically *A*_P_ = 1.42 and *A*_D_ = 1.19 during LTP and LTD, respectively. Moreover, the high symmetricity is confirmed as evidenced by superior *G* modulation during both LTP and LTD compared to the control devices. Most importantly, our proposed bifacially engineered synaptic memristor achieved recognition accuracies of 92.60% and 94.53% in CIFAR‐10 and MNIST classifications, respectively, confirming its viability for neuromorphic computing. These results underscore the experimentally validated bifacial PEAI engineering, not only overcoming the conventional limitations of perovskite memristors, but also enabling system‐level implementation of robust and high‐performance artificial synapses tailored for brain‐inspired electronics.

## Conflict of Interest

The authors declare no conflict of interest.

## Supporting information



Supporting Information

## Data Availability

The data that support the findings of this study are available in the supplementary material of this article.
