# Supplementary material for: Bifacially Engineered Perovskite‐Based Synaptic Memristors Achieve High Linearity and Symmetricity for Accurate and Robust Neuromorphic Computing
Source: Adv Sci (Weinh). 2025 Aug 20;12(42):e11489. doi: 10.1002/advs.202511489 (PMC12622500; doi:10.1002/advs.202511489)
Supplement: Supplementary file 1 — Supporting Information [file ADVS-12-e11489-s001.pdf]

Supporting Information

Bifacially Engineered Perovskite-based Synaptic Memristors Achieve High Linearity and Symmetricity for Accurate and Robust Neuromorphic Computing

Jang Woo Lee, Liang Cai, Jeong-Seok Nam, Dawoon Kim, Taehoon Kim, Sihyeok Kim, Jae Ho Lee, Cheolhwa Jang, Sungpyo Baek, Jiye Han, Kiyong Kim, Seongpil An, In Chung, Eunsang Kwon, Sungjoo Lee*, Il Jeon*

Experimental Section

Materials: All the reagents and solvents were used without further purification. Lead(II) Iodide (PbI_2 99.99%, trace metals basis) and 2-Phenylethylamine hydroiodide (PEAI) were purchased from Tokyo Chemical Industry Co., Ltd. (TCI, Tokyo, Japan). Formamidinium iodide (FAI) was purchased from Greatcell Solar Materials Pty Ltd (Australia). Methylamine hydrochloride (MACl), Isopropyl alcohol (IPA) were purchased from Sigma-Aldrich Co. Ltd. (St. Louis, MO). Dehydrated dimethylsulfoxide (DMSO, super dehydrated), dimethylformamide (DMF, super dehydrated), Chloroform (super dehydrated) and Diethyl ether (Super dehydrated) were purchased from FUJIFILM Wako Pure Chemical Co., Ltd. (Osaka, Japan).

Fabrication of Devices: Pre-patterned ITO/glass substrates (10 Ω sq^{-1}, 25 × 25 mm^2, Geomatec Co., Ltd.) were first cleaned by sonication in an ultrasonic bath with distilled water, acetone, and isopropanol for 15 min. Organic residues were then removed by UV/O_3 treatment for 30 min. The cleaned substrates were subsequently placed on a spin-coating system to begin device fabrication. A bottom layer of PEAI (20 mg/mL in isopropyl alcohol, IPA) was filtered through a 0.45 μm PVDF filter and spin-coated onto the ITO surface in two drops at 3000 rpm for 30 s. The coated substrates were then annealed at 150°C for 1 min to ensure proper film formation. Next, the perovskite layer was prepared on the PEAI-coated substrate. The precursor solution was made by dissolving FAI (395.5 mg, 2.3 mmol), PbI_2 (1060.3 mg, 2.3 mmol), and 36% MACl (55.9 mg, 0.83 mmol) in 1 mL of a 4:1 (v/v) mixture of DMF and DMSO to achieve a 2.3 mol·L^{-1} $FAPbI_3$ precursor solution. The mixture was stirred for 30 min and filtered through a 0.2 μm PTFE filter. The filtered solution was spin-coated onto the PEAI layer at 8000 rpm for 60 s. During the final 5 s of spin-coating, 1000 μL of ether was added as an antisolvent. The perovskite film was annealed at 150°C for 10 min. For the top PEAI layer, the same spin-coating procedure as for the bottom PEAI was employed. Afterward, two drops of IPA were spin-coated at 3000 rpm to remove residual PEAI from the surface, followed by annealing at 150°C for 1 min. Finally, a 100 nm thick silver (Ag) layer was deposited via thermal evaporation at a pressure of 10^{-6} Torr using a thermal evaporator. For the fabrication of control (ITO/$FAPbI_3$/Ag), ITO/PEAI/$FAPbI_3$/Ag, and ITO/$FAPbI_3$/Ag devices, the same fabrication procedure described above was followed. For each device structure, the corresponding bottom or top PEAI layer was omitted as required.

Characterizations: SEM figures were captured using S-4700 Hitachi High-Technologies CO. The UPS were measured by X-ray Photoelectron Spectrometer (NEXSA G2, UVP, Inc.). The absorption spectra of samples were measured by a UV–Vis–NIR spectrophotometer (UV 3600, Shimadzu, Japan). FTIR data was measured by infrared spectrometer (Shimadzu, IRTracer-100). The XRD was measured using the HP-Thin Film XRD (D8 ADVANCE, Bruker Corporation, USA). *I*–*V* measurements are performed by a Keithley 4200A-SCS semiconductor parameter analyzer. PL spectra were measured by Hamamatsu Quantaurus QY plus (C12354-34) with xenon lamp light source filtered by 375 nm cut-off filter. TRPL spectra were measured by Hamamatsu Quantaurus-Tau (C11367) with PLP-10, 375 nm laser source.

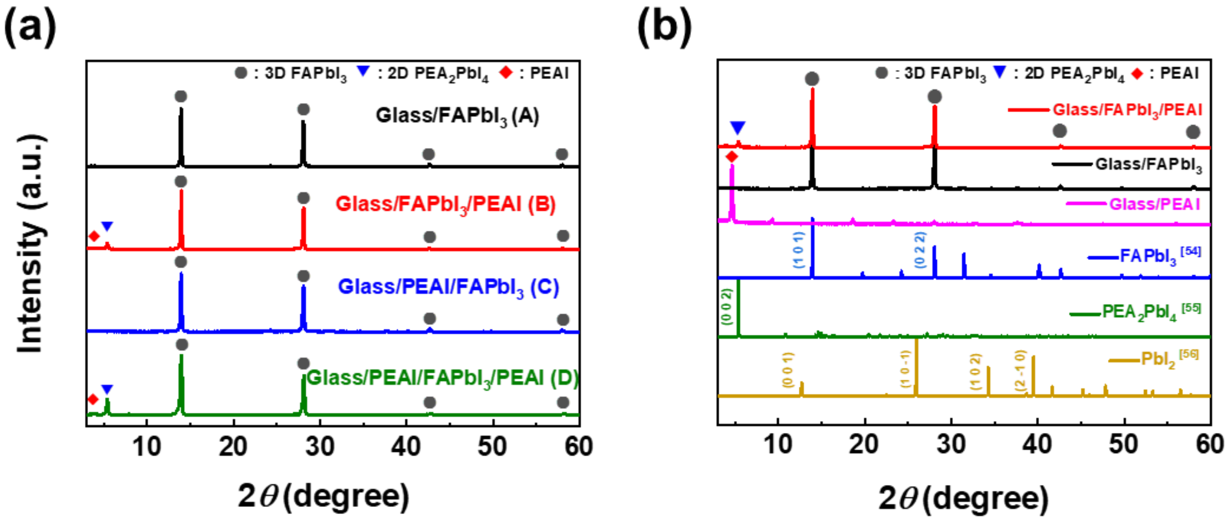

Figure S1. XRD patterns of various thin film structures. (a) XRD patterns of Glass/$FAPbI_3$, Glass/$FAPbI_3$/PEAI, Glass/PEAI/$FAPbI_3$, and Glass/PEAI/$FAPbI_3$/PEAI. (b) Comparison of the experimental XRD patterns with reference patterns of $FAPbI_3$[51], PEA_2PbI_4[52], and PbI_2[53].

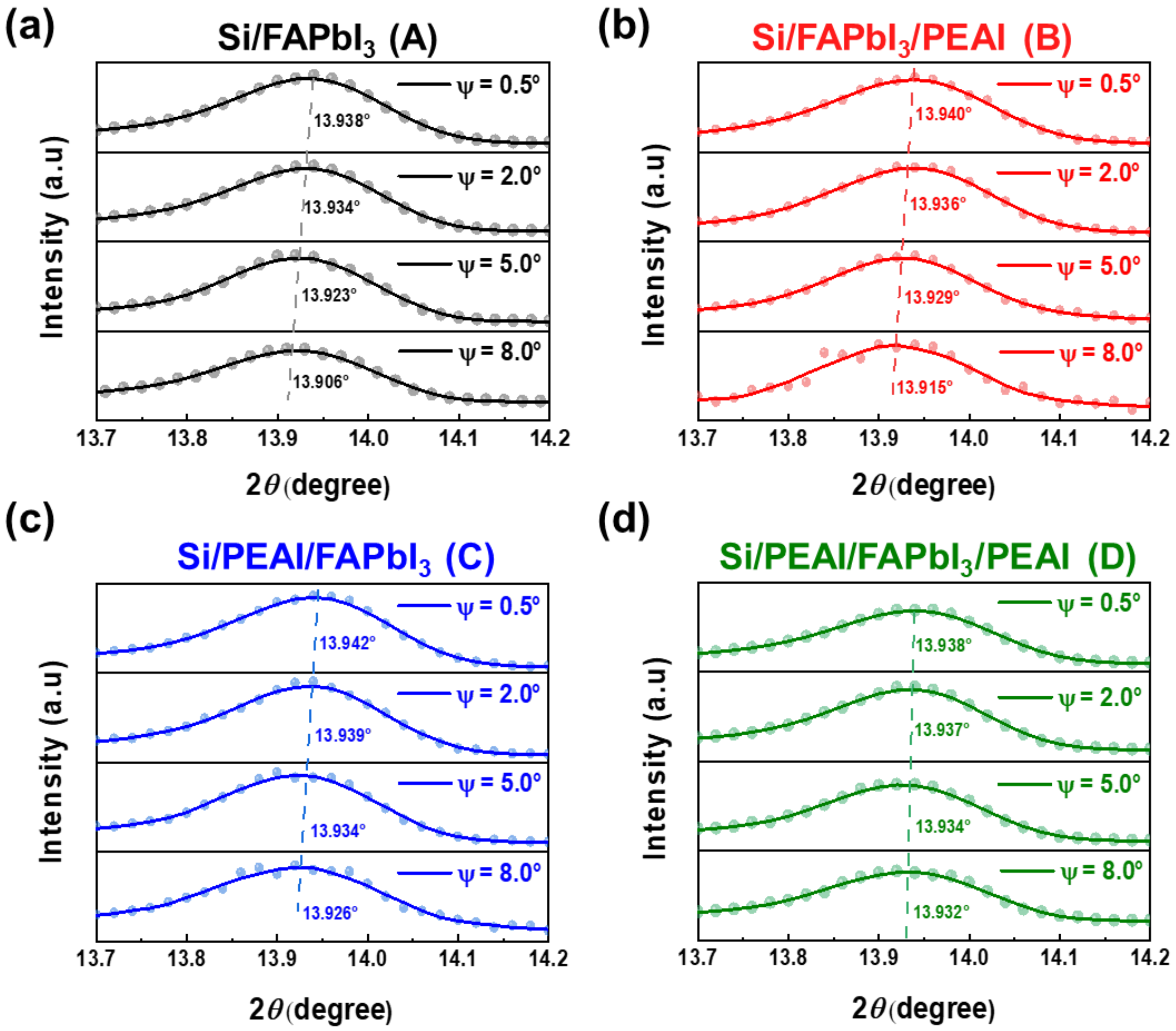

Figure S2. GIXRD ψ-scan measurements of multilayer perovskite films. GIXRD patterns at different incident angles (ψ = 0.5°, 2.0°, 5.0°, and 8.0°) for (a) Si/$FAPbI_3$, (b) Si/$FAPbI_3$/PEAI, (c) Si/PEAI/$FAPbI_3$, and (d) Si/PEAI/$FAPbI_3$/PEAI. Indicated by the consistent 2θ position (~13.9°) across ψ angles.

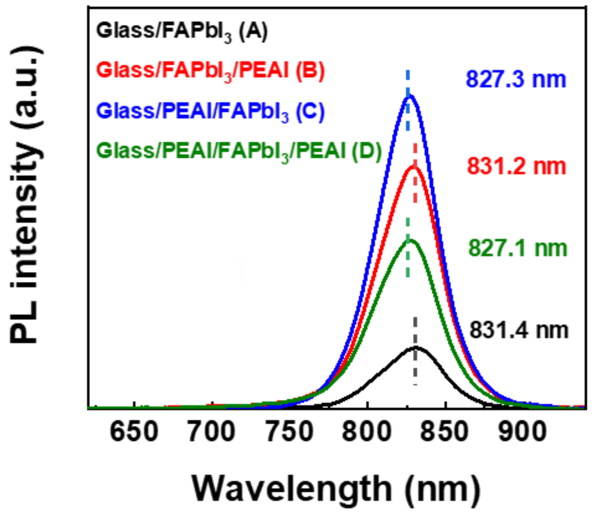

Figure S3. Steady-state PL spectra of $FAPbI_3$-based perovskite films with and without PEAI interlayers. The emission peaks are centered at 831.4 nm (Type A), 831.2 nm (Type B), 827.3 nm (Type C), and 827.1 nm (Type D).

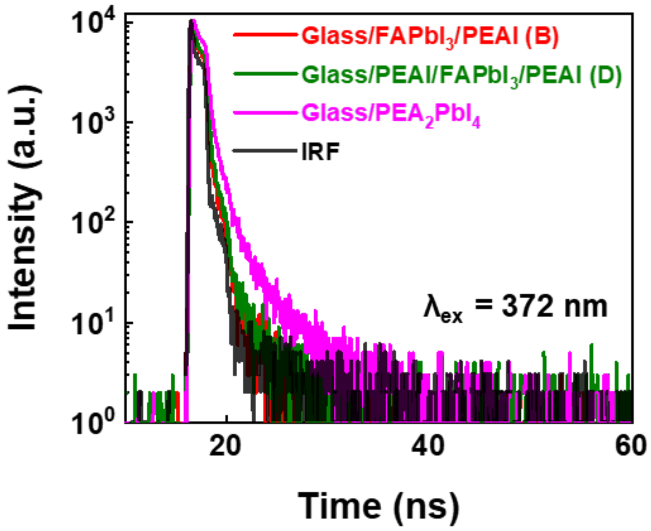

Figure S4. TRPL decay curves of Type B, Type D, and Glass/PEA_2PbI_4 films. IRF for reference (λ_{ex} = 372 nm).

Table S1. Biexponential fitting parameters for TRPL decay curves of Type B, Type D, and Glass/PEA_2PbI_4 films (λ = 525nm).

	τ_1 (ns)	τ_2 (ns)	A_1	A_2
Glass/$FAPbI_3$/PEAI (B)	0.13	0.76	4673.83	39.45
Glass/PEAI/$FAPbI_3$/PEAI (D)	0.11	0.68	5045.45	92.59
Glass/PEA_2PbI_4	0.27	1.66	2768.83	46.84

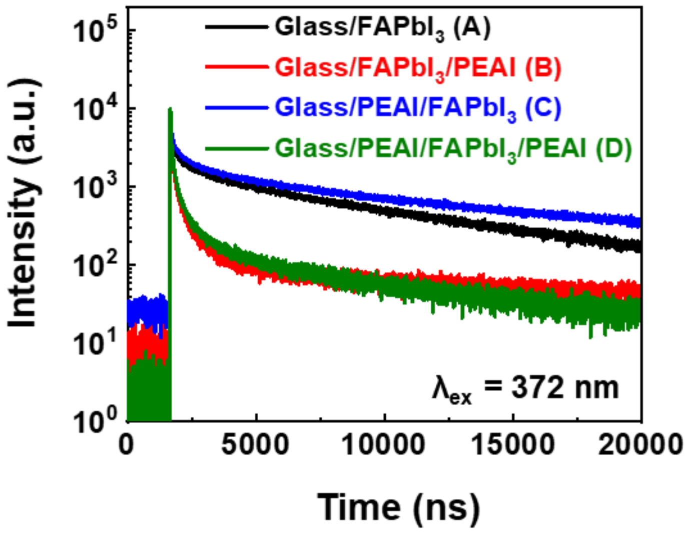

Figure S5. TRPL decay curves of Types A–D (λ_{ex} = 372 nm).

Table S2. Biexponential fitting parameters for TRPL decay curves of Types A–D (λ = 810nm).

	τ_1 (ns)	τ_2 (ns)	A_1	A_2
Glass/ $FAPbI_3$ (A)	19.05	160.70	7981.59	2408.89
Glass/$FAPbI_3$/PEAI (B)	21.57	132.93	7321.33	3136.93
Glass/PEAI/$FAPbI_3$ (C)	15.58	187.13	8522.09	1797.17
Glass/PEAI/$FAPbI_3$/PEAI (D)	22.64	149.15	7077.44	3062.94

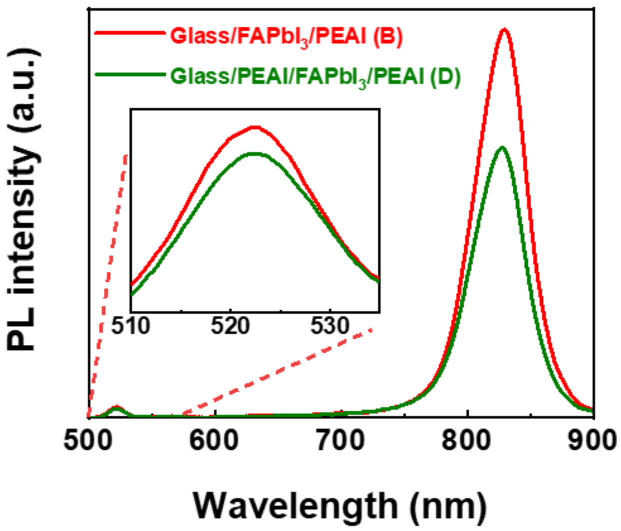

Figure S6. Steady-state PL spectra of Type B and Type D. The main PL peaks around 830 nm correspond to $FAPbI_3$. The inset highlights a weaker emission feature in the 510–530 nm range, attributed to the PEA_2PbI_4.

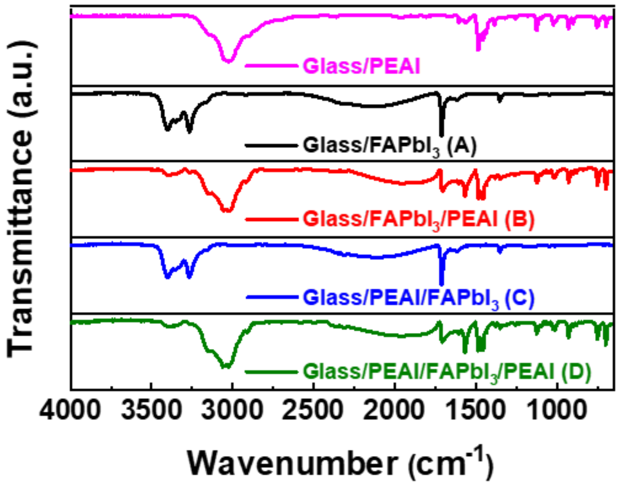

Figure S7. FTIR spectra of Glass/PEAI and MHP films of Types A–D measured in the range of 800–4000 cm^{-1}.

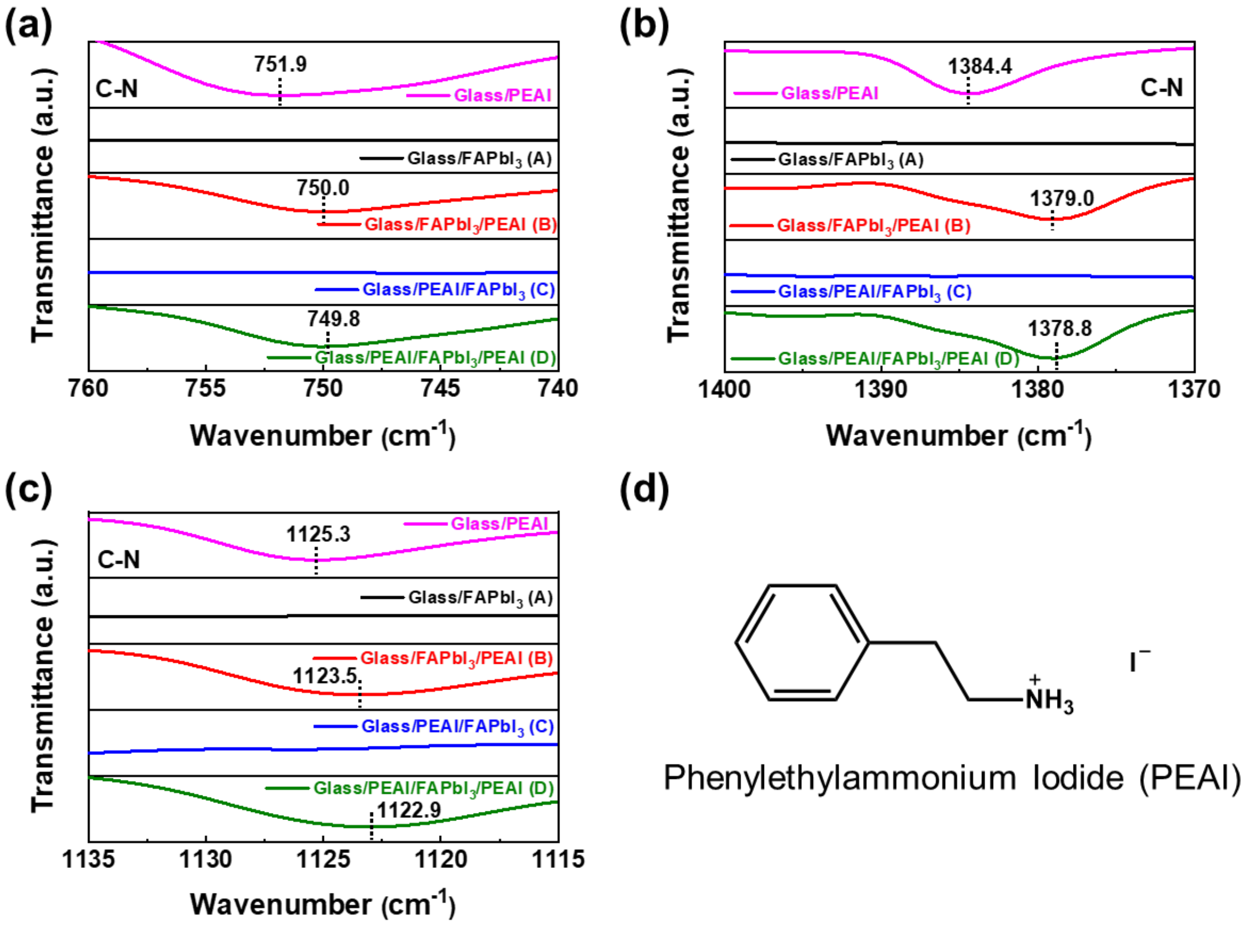

Figure S8. FTIR spectra of Glass/PEAI and MHP films of Types A–D measured in the range of (a) 740–760 cm^{-1}, (b) 1370–1400 cm^{-1}, (c) 1115–1135 cm^{-1}. (d) Chemical structure of PEAI.

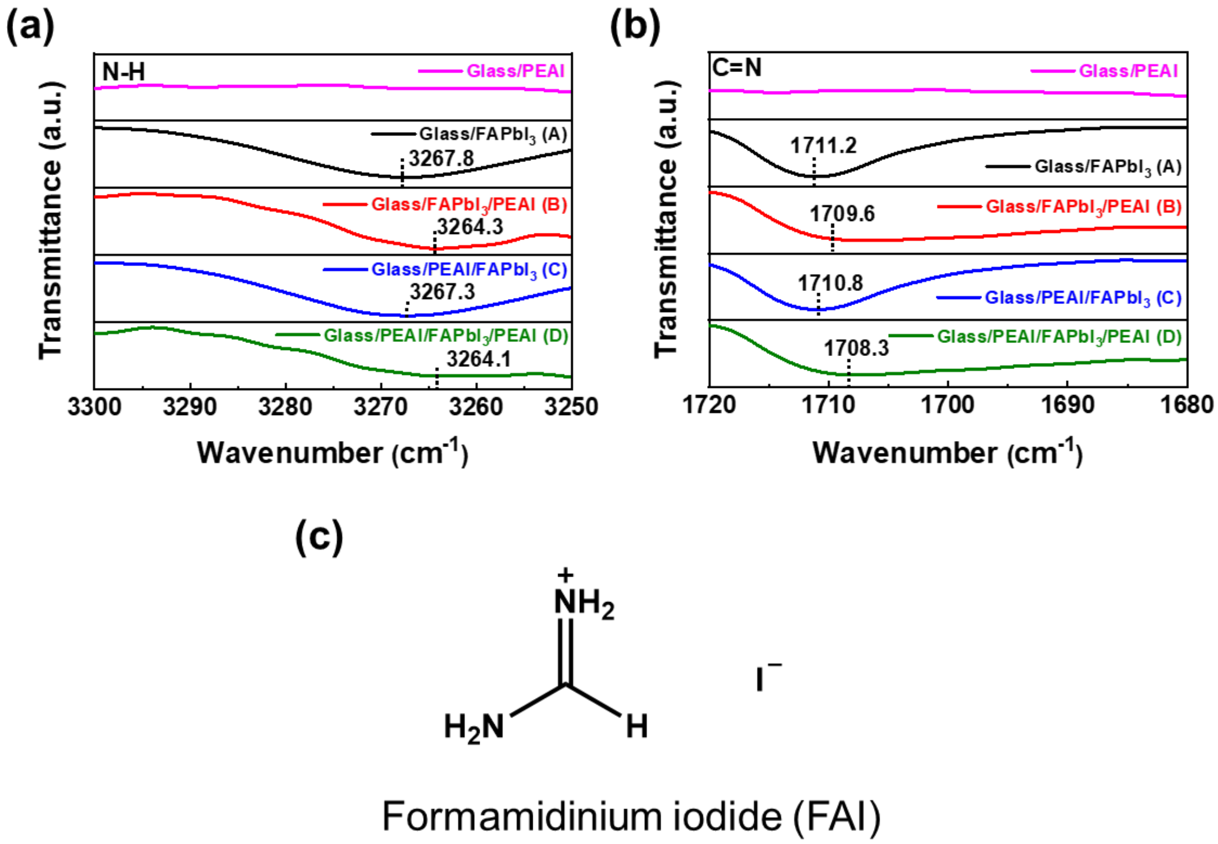

Figure S9. FTIR transmittance spectra of Glass/PEAI and MHP films of Types A–D measured in the range of (a) 3250-3300 cm^{-1}, (b) 1680-1720 cm^{-1}. (c) Chemical structure of FAI.

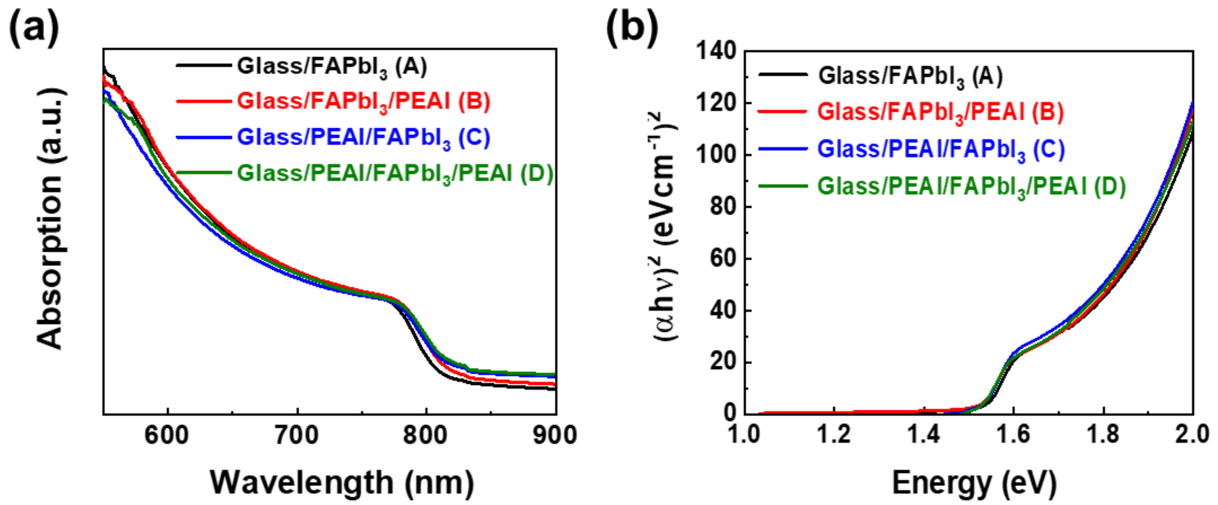

Figure S10. (a) UV–vis–NIR absorption spectra and (b) Tauc plots for MHP films of Types A–D.

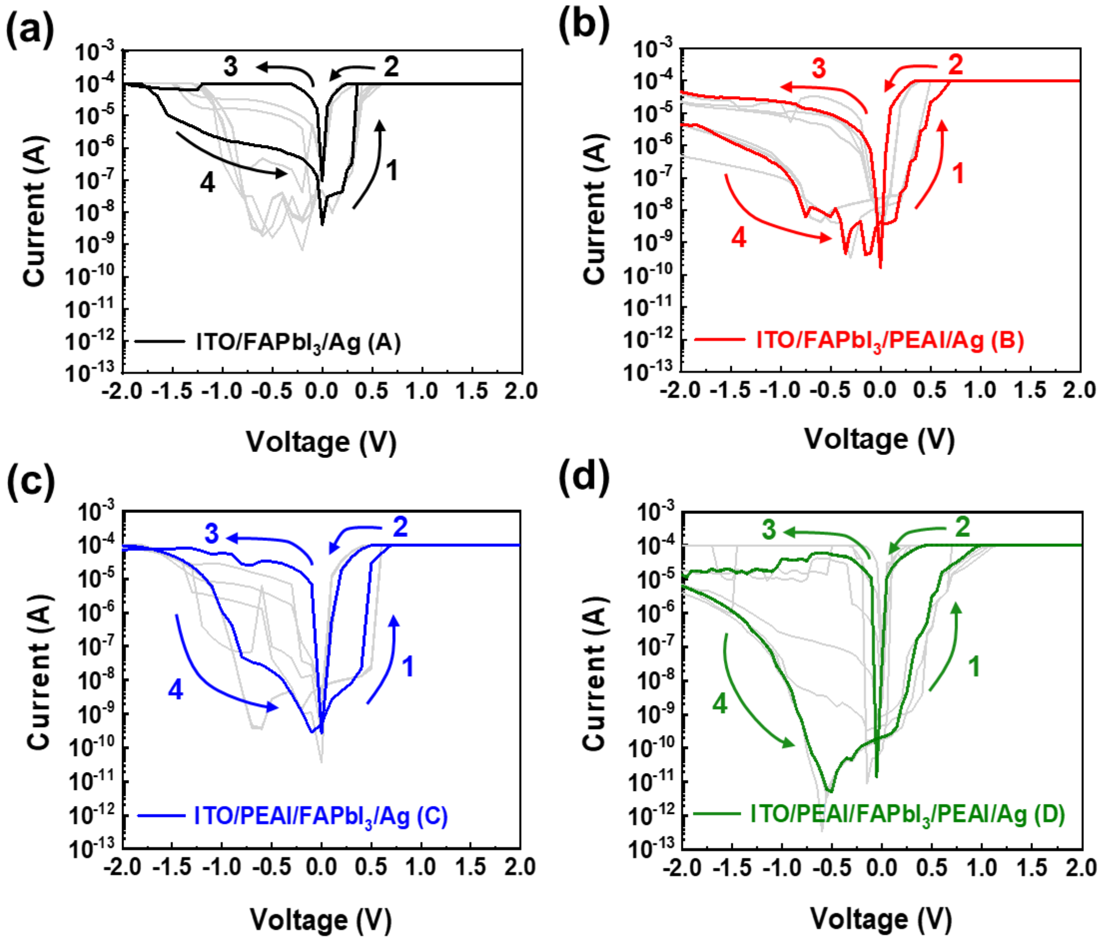

Figure S11. *I–V* characteristics comparison of the four types of MHP memristors for (a) Type A, (b) Type B, (c) Type C, and (d) Type D. Each device was tested over 100 cycles. Gray lines represent every 20th cycle, while colored lines indicate the pristine state.

Table S3. I_{on}, I_{off} and I_{on}/I_{off} ratios extracted from the *I–V* characteristics at a bias voltage of 0.15 V for devices with the following structures (The compliance current is set to be 10^{-4} A).

	I_{on} (A)	I_{off} (A)	I_{on} / I_{off}
ITO/$FAPbI_3$/Ag (A)	8.23×10^{-5}	4.39×10^{-8}	1.87×10^{3}
ITO/$FAPbI_3$/PEAI/Ag (B)	6.92×10^{-5}	2.23×10^{-8}	3.10×10^{3}
ITO/PEAI/$FAPbI_3$/Ag (C)	4.34×10^{-5}	8.32×10^{-9}	5.22×10^{3}
ITO/PEAI/$FAPbI_3$/PEAI/Ag (D)	3.19×10^{-5}	4.15×10^{-10}	7.69×10^{4}

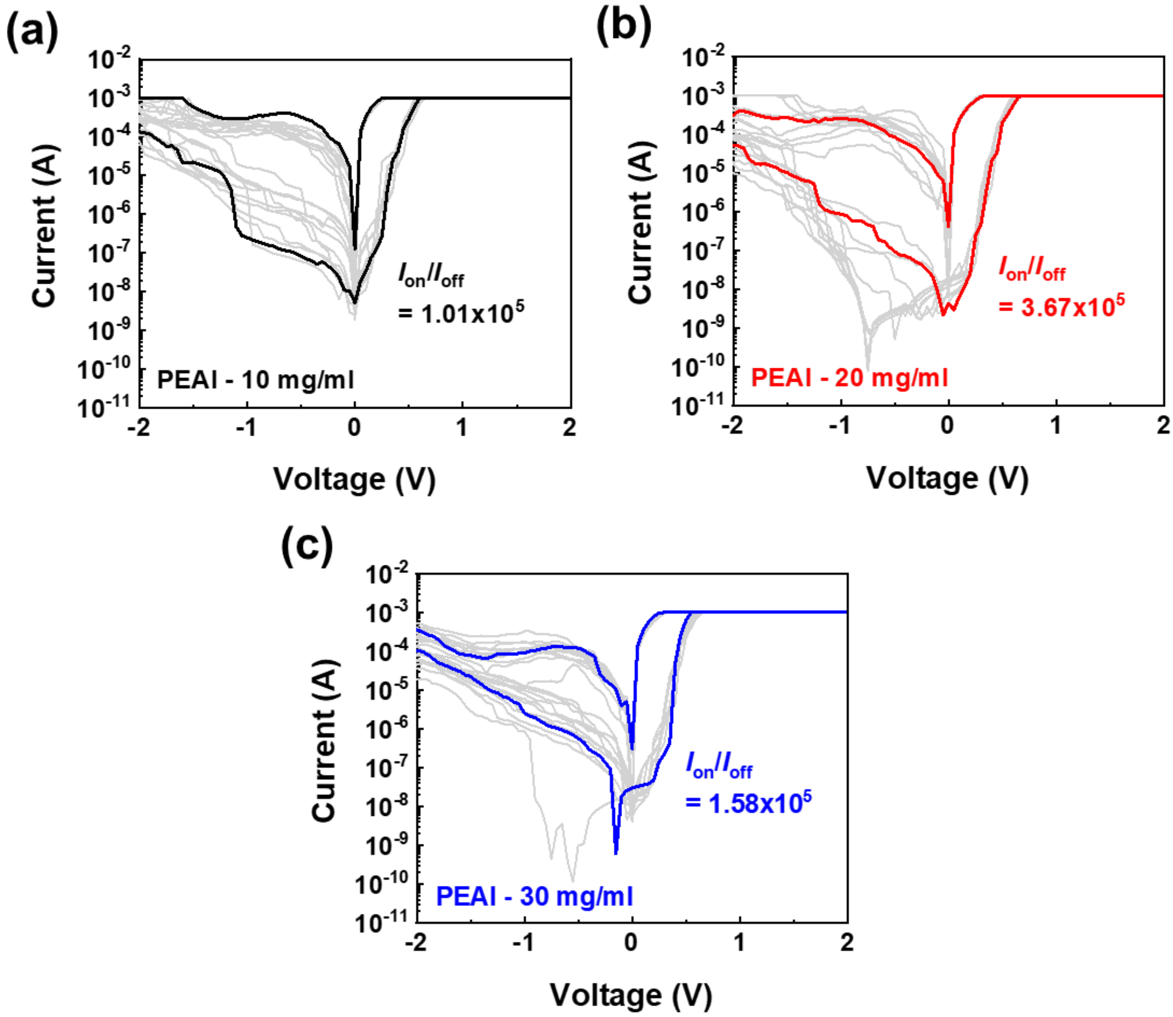

Figure S12. Comparison of the *I–V* characteristics of memristor devices with varying PEAI concentrations: (a) 10 mg/mL, (b) 20 mg/mL, and (c) 30 mg/mL. The device structure is Type D: ITO/PEAI/$FAPbI_3$/PEAI/Ag. The compliance current is set to be 10^{-3} A

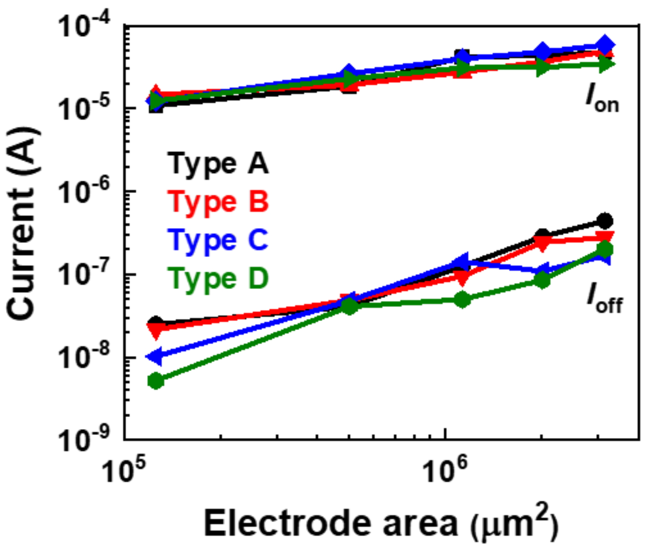

Figure S13. Measured I_{on} and I_{off} of four types of MHP-based memristors with respect to the electrode area.

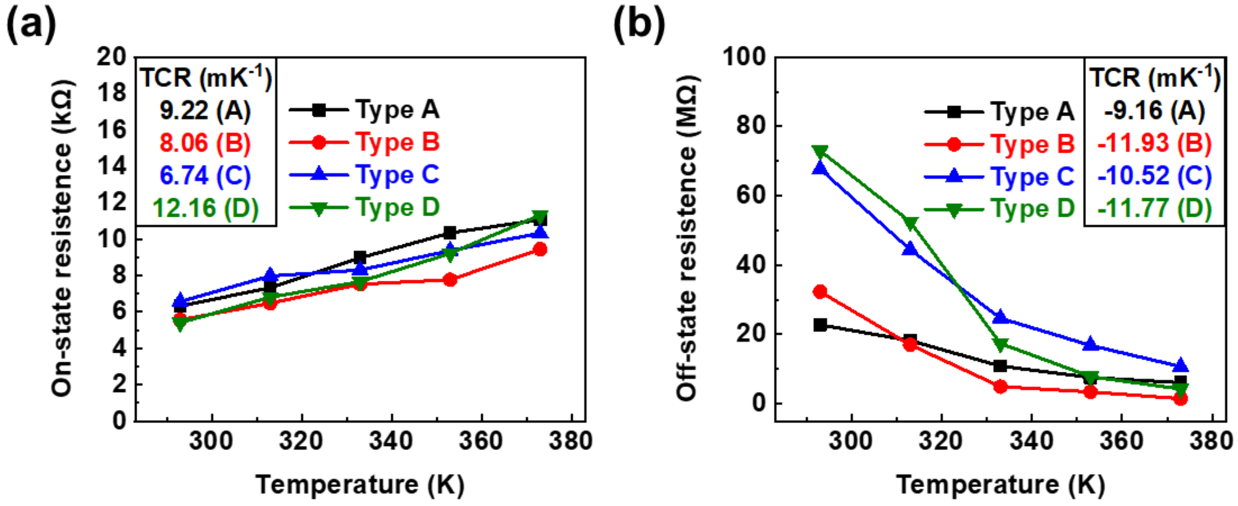

Figure S14. Temperature-dependent R characteristics in both on-state and off-state.

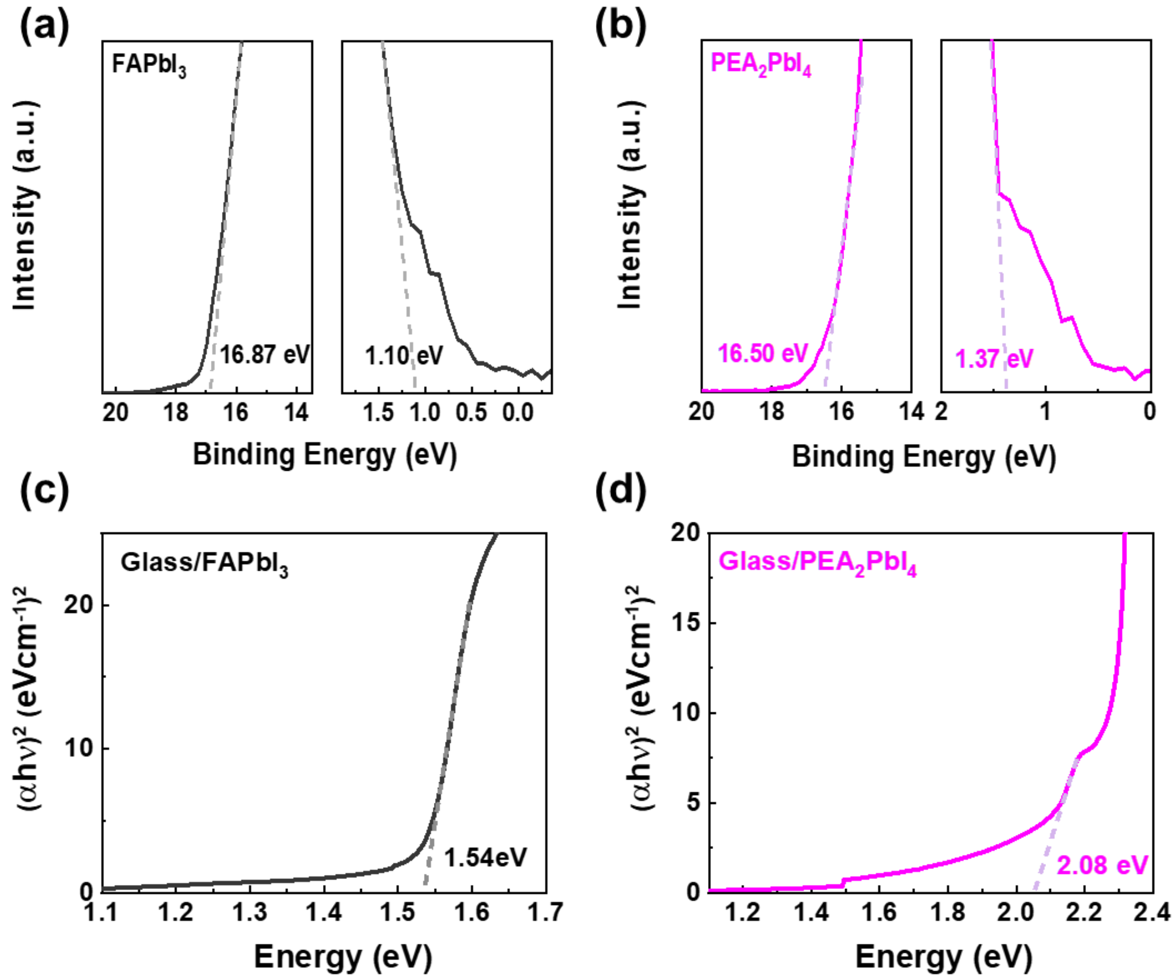

Figure S15. UPS and UV–vis absorption spectra of $FAPbI_3$ and PEA_2PbI_4 perovskite films. UPS spectra of (a) $FAPbI_3$ and (b) PEA_2PbI_4 films deposited on Si substrates. Tauc plots from UV–vis absorption spectra of (c) Glass/$FAPbI_3$ and (d) Glass/PEA_2PbI_4 films used to investigate the optical bandgap.

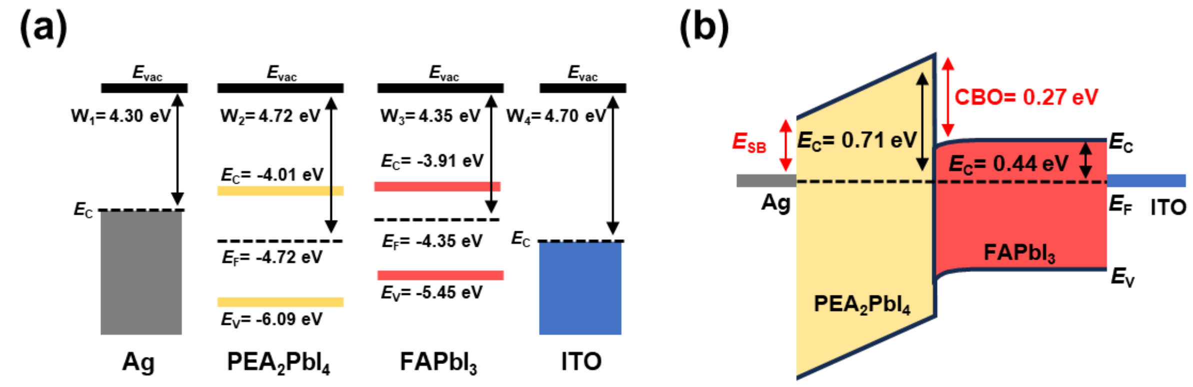

Figure S16. (a) Energy level alignment of Ag, PEA_2PbI_4, $FAPbI_3$, and ITO. The E_c, E_v, E_F, and work-function (WF) values were extracted from UPS and UV-vis measurement. (b) Fermi level aligned energy band diagram of the proposed synaptic memristor, Type D.

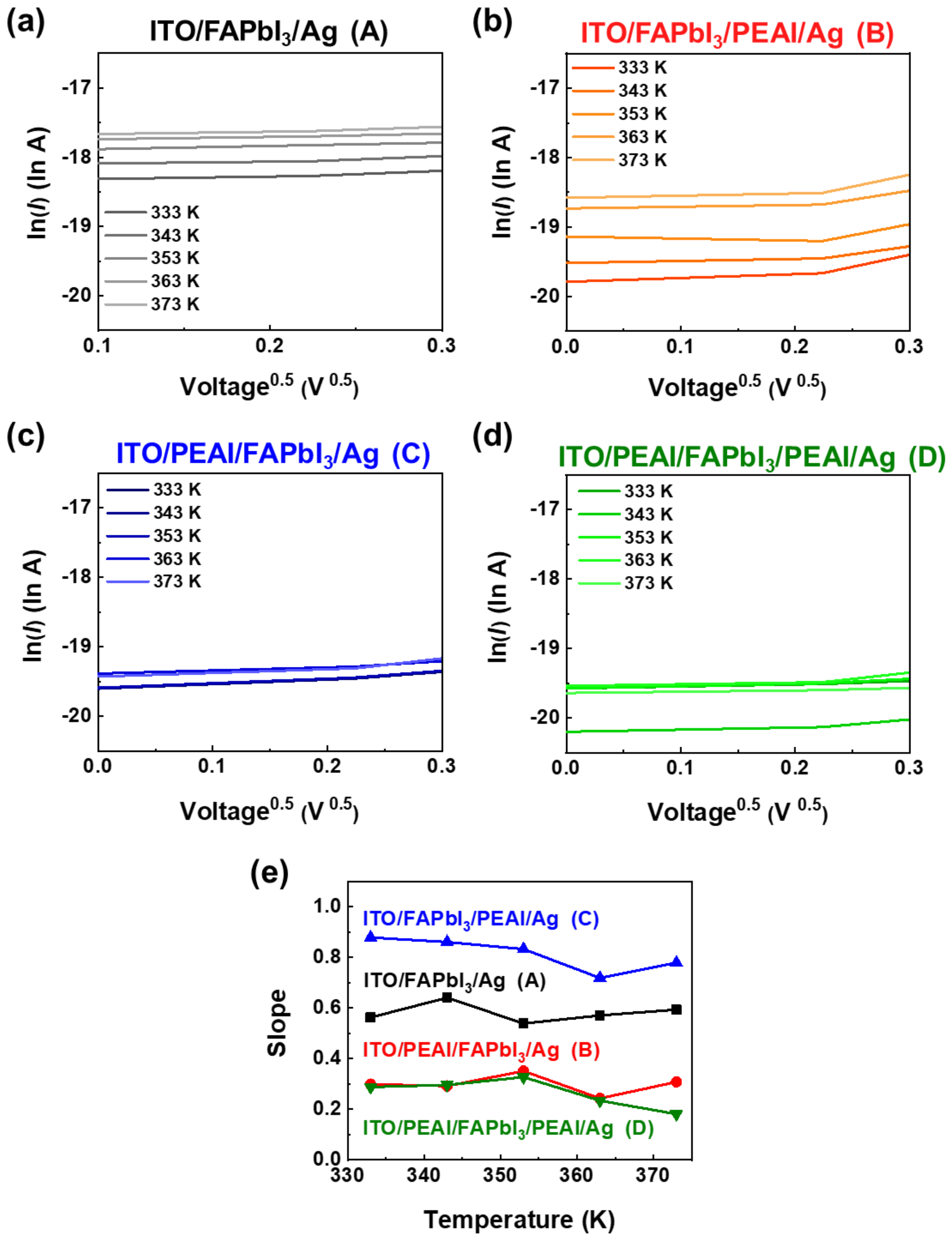

Figure S17. Ln(I)–$V^{0.5}$ plots of perovskite memristor devices in the I_{off} region, measured at temperatures ranging from 333 K to 373 K within the $V^{0.5}$ range of 0.1–0.3. Device structure includes followings: (a) Type A, (b) Type B, (c) Type C, and (d) Type D. (e) Corresponding slopes of the ln(I)–$V^{0.5}$ curves for all Types A–D memristors as a function of temperature (333–373 K).

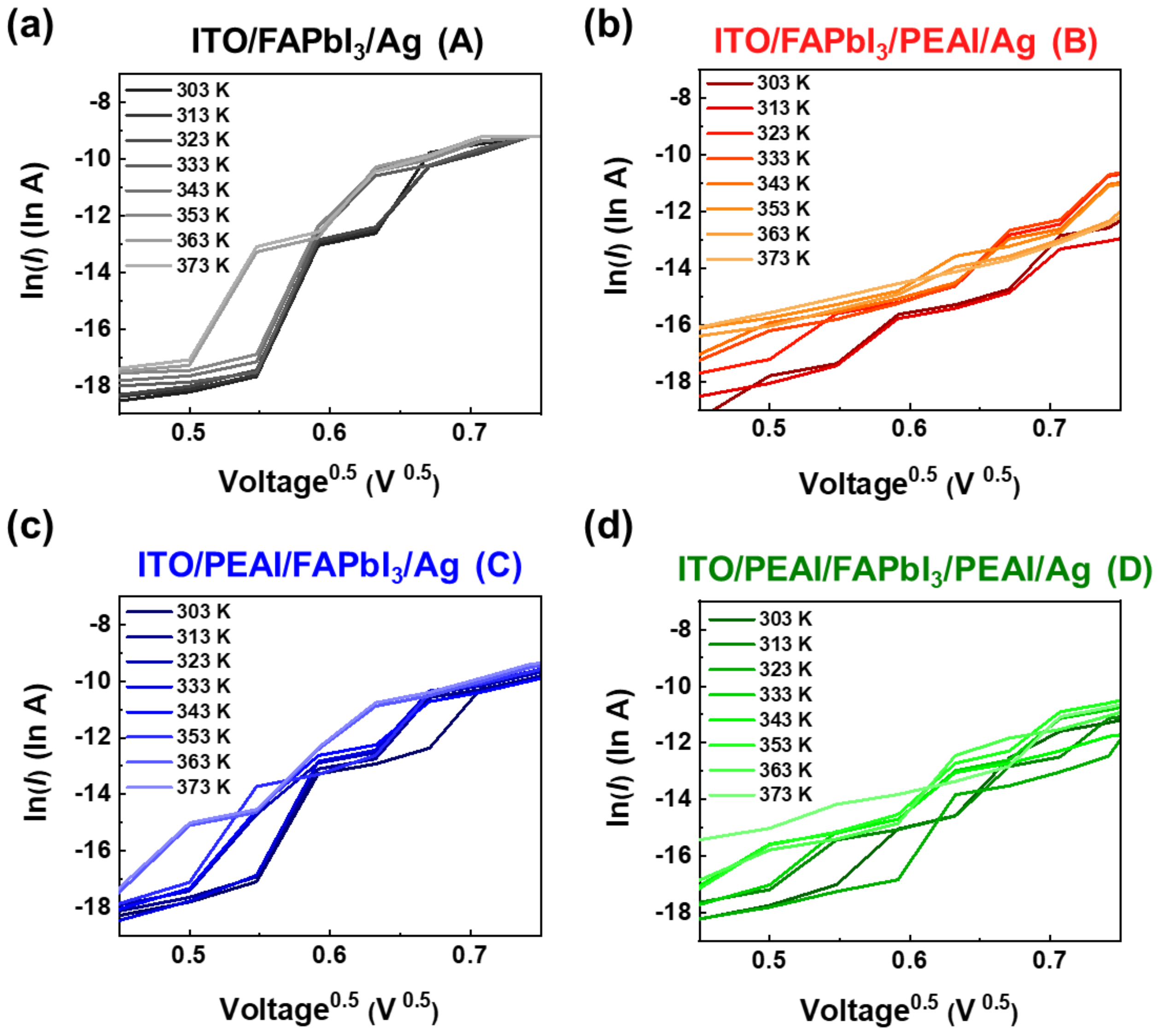

Figure S18. Ln (I)–$V^{0.5}$ plots of perovskite memristor devices in the switching region, measured at temperatures ranging from 303 K to 373 K within the $V^{0.5}$ range of 0.55–0.75. Device structure include: (a) Type A, (b) Type B, (c) Type C, and (d) Type D.

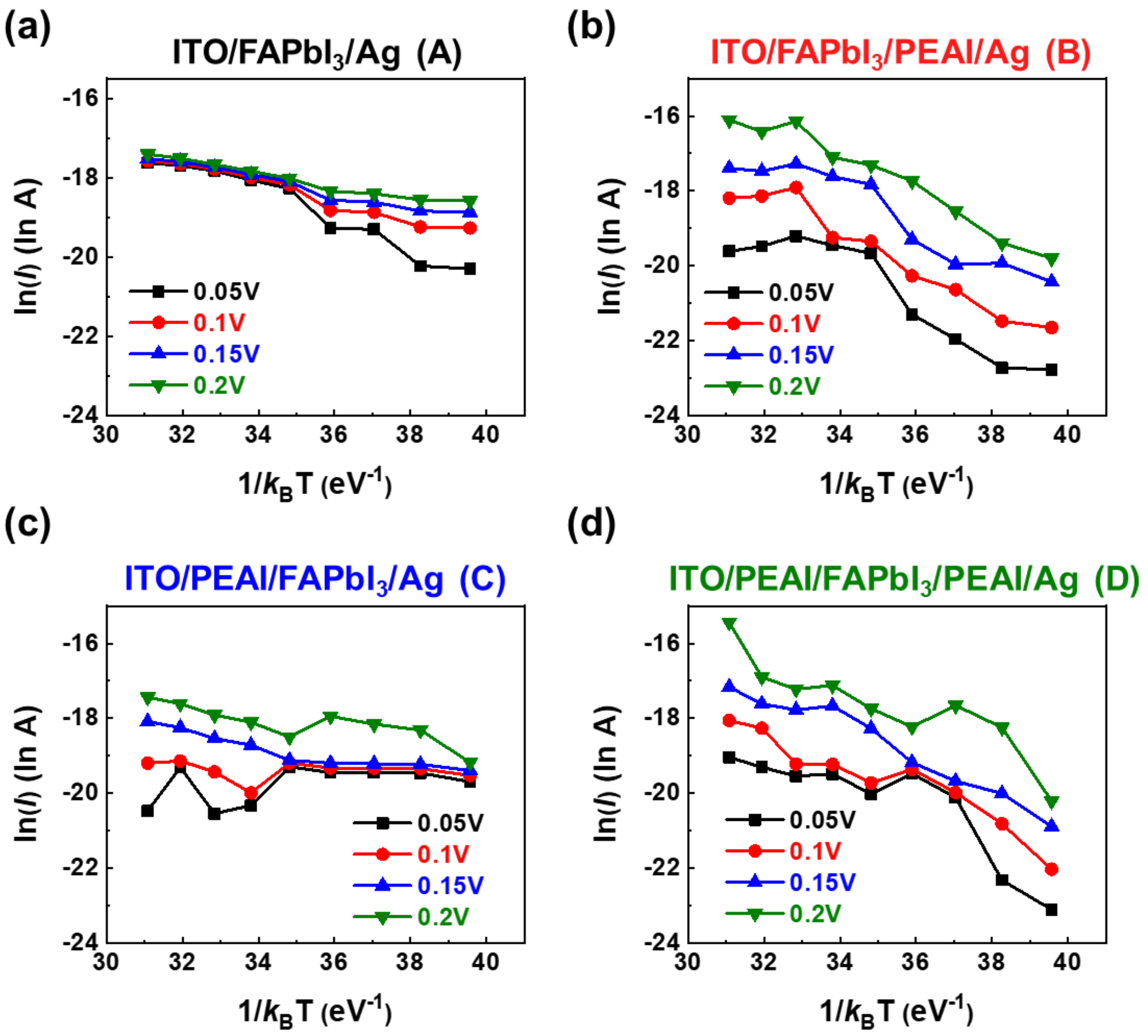

Figure S19. Ln (I) vs. 1/k_BT plot of all Types A–D devices following Arrhenius equation extracted from the voltages at 0.05V, 0.1V, 0.15V, and 0.2V respectively. (a) Type A, (b) Type B, (c) Type C, and (d) Type D.

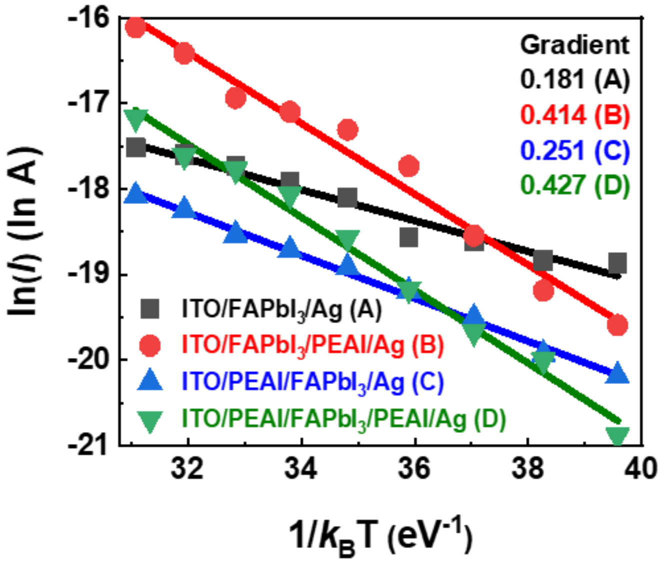

Figure S20. Calculated gradients from the ln (I) vs. $1/k_BT$ plots of all Types A–D devices extracted from the I_{off} regime (0.15 V). The gradients for Type A, B, C, and D are 0.181, 0.414, 0.251, and 0.427, respectively.

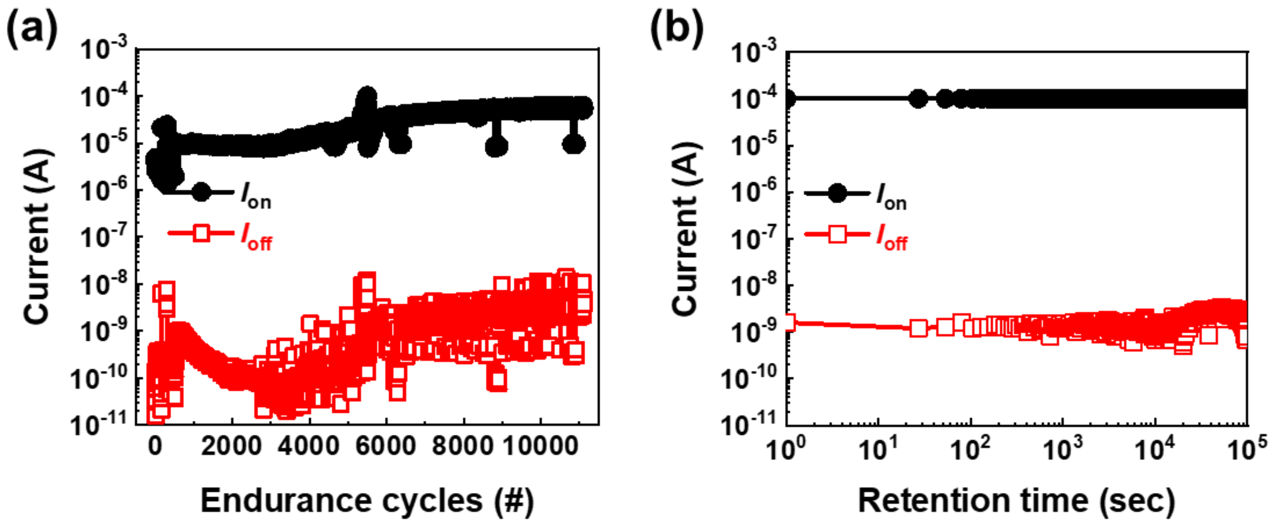

Figure S21. (a) Endurance cycles of Type D over 11,000 cycles, with sustained I_{on}/I_{off} ratio around ~10^5. (b) Retention characteristic of Type D satisfying over 10^5 s.

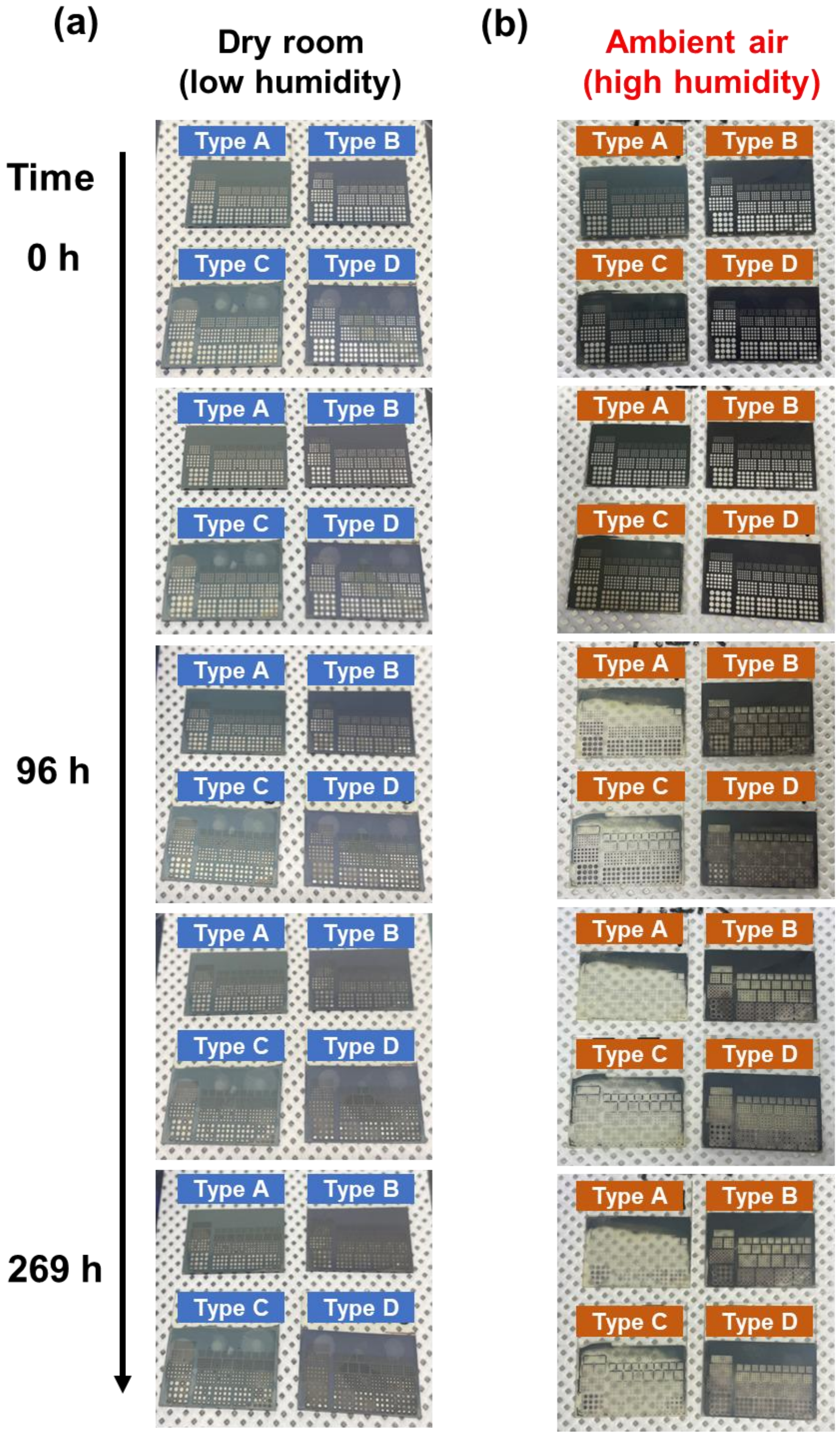

Figure S22. Analysis of the surface morphology of Types A–D over a period of 269 h (~11 day) under different environmental conditions: (a) Dry room (low humidity) and (b) Ambient air (high humidity). The comparison highlights the impact of humidity on the morphological stability of the devices.

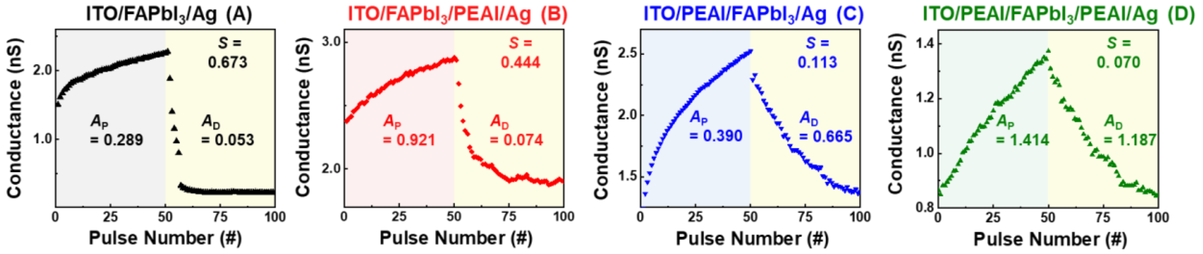

Figure S23. Measured LTP and LTD curves of Types A–D showing the synaptic behavior.

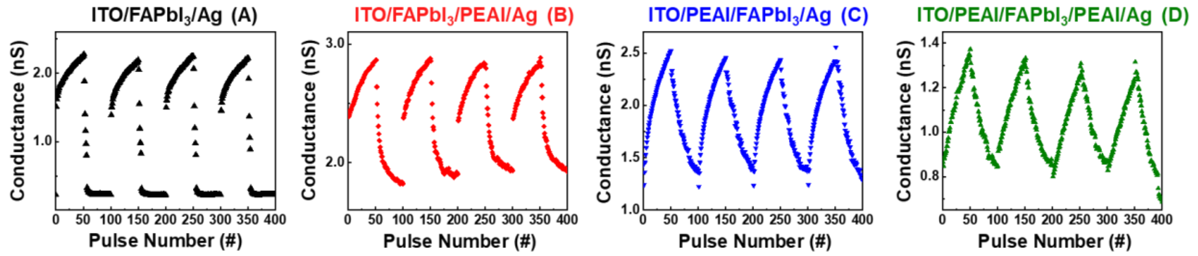

Figure S24. Cycle-to-cycle measurements of LTP and LTD curves for Types A–D, demonstrating the reproducibility and synaptic behavior.

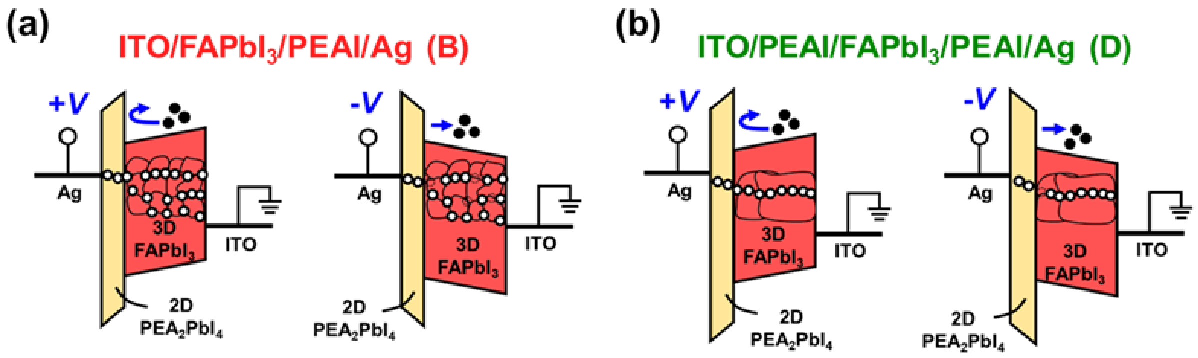

Figure S25. Energy band diagrams depicting the formation and rupture of CFs in (a) Type B and (b) Type D.

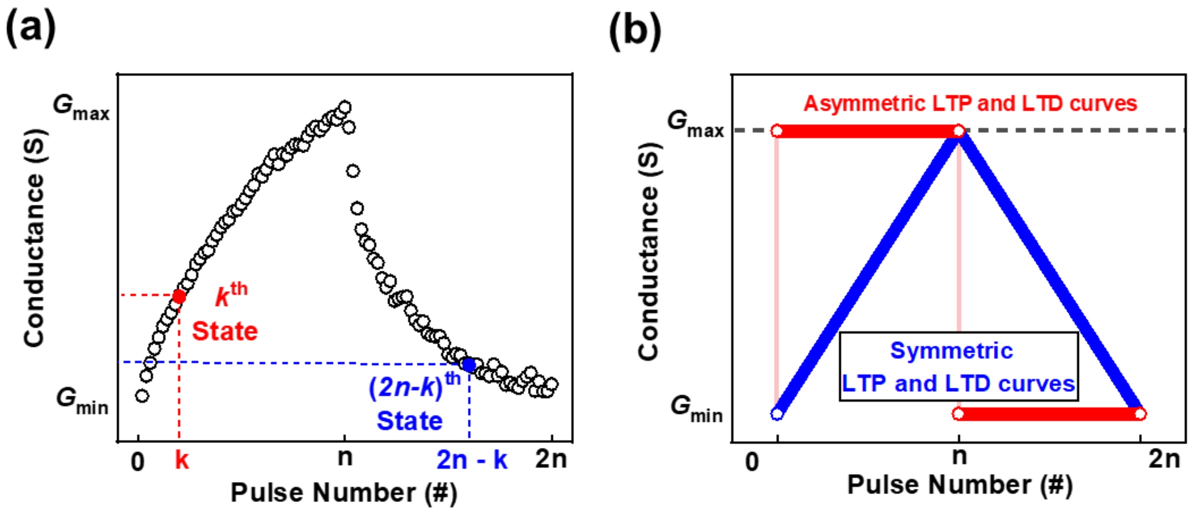

Figure S26. (a) LTP and LTD curves comprising 2n G states, highlighting the kth G_{LTP} (red dot) and the symmetrically paired $(2n\text{-}k)^{th}$ G_{LTD} (blue dot). (b) Illustration of LTP and LTD curves in two extreme cases: maximally asymmetric ($S = G_{max}$ - G_{min}) and perfectly symmetric ($S = 0$) in LTP and LTD curves.

In **Figure S26a**, LTP and LTD curves of the synaptic memristors, highlighting its kth G_{LTP} (red dot), which is paired with the $(2n\text{-}k)^{th}$ G_{LTD} (blue dot) for symmetricity evaluation purpose. Also, we defined the S by calculating the root mean square error between the G_{LTP} and G_{LTD} as follows:

$$S = \sum_{k=1}^{k=n} \frac{(G_N(k) - G_N(2n-k))^2}{n}$$

Where $G_N(k) = \frac{G(k) - G_{min}}{G_{max} - G_{min}}$

Figure S26b shows two extreme cases of LTP and LTD curves in terms of symmetricity: maximally asymmetric ($S = G_{max}$ - G_{min}) and perfectly symmetric ($S = 0$) in LTP and LTD curves.

Table S4. Summary of linearity and symmetricity metrics for Types A−D; A_P, α_P A_D, α_D, S, and G_{max}/G_{min} during LTP and LTD.

	A_P	α_P	A_D	α_D	G_{max}/G_{min}	S
ITO/$FAPbI_3$/Ag (A)	0.289	3.79	0.053	7.98	3.005	0.673
ITO/$FAPbI_3$/PEAI/Ag (B)	0.921	1.35	0.074	7.41	2.230	0.444
ITO/PEAI/$FAPbI_3$/Ag (C)	0.390	2.98	0.665	1.84	2.922	0.113
ITO/PEAI/$FAPbI_3$/PEAI/Ag (D)	1.415	0.89	1.187	1.05	1.920	0.070
Ideal synapse	-	0	-	0	>20	0

Table S5. Benchmarks of CIFAR-10 and MNIST simulation results based on MHP synaptic memristors predicted by DNN+NeuroSim v2.1 and MLP+NeuroSim v3.0 simulator, respectively.

Device Structure	A_P / A_D	Pulse condition	CIFAR-10 Accuracy (%)	MNIST Accuracy (%)	Year
**ITO/PEAI/$FAPbI_3$/PEAI/Ag**	**1.42 / 1.19**	**± 0.6 V / 100 ms**	**92.60**	**94.53**	**2025 (This work)**
Al/$MAPbBr_3$/ITO	-	± 3 V / 500 ms	-	80.80	2018[108]
ITO/$MAPbI_3$/Ag	-	± 0.3 V / 400 ms	-	82.70	2019[33]
ITO/KI-$MAPbI_3$/Au	-	± 1 V / 2 ms	-	84.20	2021[34]
ITO/PEDOT:PSS/$FABi_3I_{10}$+$FA_3Bi_2I_9$/Ag	2.42/0.45	± 0.2 V / 300 µs	87.00	92.90	2022[23]
Si/AlO_x/InO_x/$FA_{0.85}MA_{0.15}Pb(I_{0.85}Br_{0.15})_3$/ZnO	-	-	90.00	-	2023[109]
Si/SiO_2/$(EATPCN)_2PbI_4$/Pentacene/Au	0.92/2.58	-	89.00	94.80	2023[110]
ITO/b-PEAI/ZnO/$Cs_{1-x}FA_xPbBr_3$/V_2O_{5-y}/Al	0.60 / 0.22	± 2 V / 300 ms	-	89.08	2024[35]
Ag/δ-$FAPbI_3$/SnO_2/ITO	1.67 / 0.29	± 0.7 V / 500 µs	-	94.04	2024[32]
Au/$CsPbBr_3$/Au	-	± 10 V / 45 ms	-	93.11	2024[111]
FTO/$BA_{0.15}MA_{0.85}PbI_3$/Ag	-	± 0.5 V / 2.5 ms	-	94.80	2024[31]
ITO/$BDAMA_{n-1}Pb_nI_{3n+1}$/PMMA/Au	-	± 1.5 V / 500 µs	92.29	98.75	2025[6]
Au/PMMA/$Cs_3Bi_2I_9$/PMMA/Au	0.83 / 1.14	± 0.5 V / 50 ns	91.98	88.19	2025[42]
Paper/PMMA/Ag/$BDAPbI_4$/ PMMA/ graphite	0.51 / 0.56	± 0.5 V / 10 ms	83.00	-	2025[112]

Supporting Note S1

Feed-forward propagation: In the 1st convolutional operation, the input image pixels are represented as V signals and are convolved with 128 kernels consisting of synaptic weights (W), thereby generating multiple feature maps containing I signals ($I = W \times V$).[103,106] These I signals are then passed through a rectified linear unit (ReLU) activation function and transformed into V signals, which are propagated to the subsequent (2nd) convolutional layer. After completing the convolutional operation in the 2nd layer in the same manner as in the 1st layer, the resulting V signals are transmitted to the subsequent pooling layer. In this pooling layer, a down-sampling operation is performed by aggregating local regions of the feature maps using a max-pooling function, thereby reducing the spatial resolution while retaining the most salient and discriminative features. This process not only enhances computational efficiency but also enables the extraction of higher-level feature representations as the outputs are propagated to the next convolutional block. Following this sequence, a total of six convolutional operations and three pooling operations are performed, after which the resulting 1D-flattened vector of size 8192 is fed into a fully connected neural network (FCNN). The FCNN consists of two hidden layers, with weight matrix sizes of 8192 × 1024 and 1024 × 10, respectively. Consequently, the 1D-flattened 8192 input data undergoes weighted-sum (or multiply-accumulate, MAC) operations, *i.e.*, $I = \Sigma W \times V$ and activation processes through the 1st and 2nd hidden layers in FCNN, ultimately producing the predicted label corresponding to one of the ten classes of CIFAR-10.

Backpropagation: To minimize errors (δ) during the feed-forward propagation, the difference between the true label values (K) of the training images and the output voltage signals (V_o) generated by the FCNN is computed ($\delta = K - V_o$). Synaptic weights are subsequently updated in a layer-wise manner, propagating from the output layer back to the first convolutional layer, to reduce these errors.
